# Computational implementation of a tunable multicellular memory circuit for engineered eukaryotic consortia

**DOI:** 10.3389/fphys.2015.00281

**Published:** 2015-10-09

**Authors:** Josep Sardanyés, Adriano Bonforti, Nuria Conde, Ricard Solé, Javier Macia

**Affiliations:** ^1^ICREA-Complex Systems Lab, Department of Experimental and Health Sciences, Universitat Pompeu FabraBarcelona, Spain; ^2^Institut de Biologia Evolutiva, CSIC-UPFBarcelona, Spain; ^3^Santa Fe InstituteSanta Fe, NM, USA

**Keywords:** computational modeling, eukaryotic memory circuits, flip-flop, multicellular circuits, synthetic biology

## Abstract

Cells are complex machines capable of processing information by means of an entangled network of molecular interactions. A crucial component of these decision-making systems is the presence of memory and this is also a specially relevant target of engineered synthetic systems. A classic example of memory devices is a 1-bit memory element known as the flip-flop. Such system can be in principle designed using a single-cell implementation, but a direct mapping between standard circuit design and a living circuit can be cumbersome. Here we present a novel computational implementation of a 1-bit memory device using a reliable multicellular design able to behave as a set-reset flip-flop that could be implemented in yeast cells. The dynamics of the proposed synthetic circuit is investigated with a mathematical model using biologically-meaningful parameters. The circuit is shown to behave as a flip-flop in a wide range of parameter values. The repression strength for the NOT logics is shown to be crucial to obtain a good flip-flop signal. Our model also shows that the circuit can be externally tuned to achieve different memory states and dynamics, such as persistent and transient memory. We have characterized the parameter domains for robust memory storage and retrieval as well as the corresponding time response dynamics.

## 1. Introduction

Developing living devices that can perform non-trivial decisions is one of the major challenges of synthetic biology (Purnick and Weiss, 2009). By introducing new components, changing existing paths or allowing novel forms of signal reception, engineered cells can cope with novel decision-making scenarios. Among the requirements to achieve such goal, memory is specially relevant to store and retrieve past events. Biological memory implies a sustained cellular response to a transient stimulus (Burrill and Silver, 2010) and it pervades the potential for adaptation and learning.

The qualitative characterization of how a cell might achieve biological memory through its transcriptional circuitry was determined nearly 50 years ago by Monod and Jacob (1961). Despite this early work, the quantitative understanding of these circuits has been achieved recently (Alon, 2006). Inspired in this knowledge, synthetic memory devices have been developed at different scales (Burrill and Silver, 2010; Inniss and Silver, 2013) during the last decade. In an early work, Gardner et al. (2000), developed a toggle switch in *E. coli*. This system is based on a two mutual repressors architecture having two stable steady states. In one state, the gene for the first repressor is turned on, and the synthesis of the second repressor is therefore turned off. The absence of the second repressor allowed maintaining the stable state, where the presence of the first one acted as an indirect activator of its own synthesis by repressing its own repressor. In the other steady state, the second repressor is present and the first is absent, following the same logic. By addition of external inducers the device is able to change the stable state. A different architecture was implemented in yeast (Ajo-Franklin et al., 2007) using a transcriptional positive feedback with sensitivity to cell growth. Here, memory was sustained by an autoregulatory transcriptional positive feedback. Other devices have been explored, including conditional memory systems (Fritz et al., 2007), implementations based on the expression of specific recombinases in bacteria (Siuti et al., 2013) or switchable memories using DNA biochemistry *in vitro* (Padirac et al., 2012; Inniss and Silver, 2013).

The *in vivo* implementation of different subsets of synthetic circuits can be seriously limited by several constraints. A major problem is related to the presence of undesired interactions between added components and the existing cellular machinery (Kwok, 2010). Several approximations have been explored in order to overcome these limitations. Among others, an implementation based on cellular consortia gives some engineering and robustness advantages (Macia et al., 2002; Li and You, 2011; Tamsir et al., 2011; Solé and Macia, 2013; Macia and Solé, 2014). It provides a desirable compartmentalization that limits cross-talk (cell-to-cell communication), reducing the required engineering in each cell and exploiting the reuse of molecular toolkit components.

The main goal of the present work is to explore the potential of multicellular computation for implementing sequential logic circuits in living cells and cellular consortia, i.e., circuits in which feedback connections are present. More specifically, we focus on 1-bit memory devices as a relevant case of study. Other theoretical studies have analyzed the problem of how to design a single-cell standard flip-flop in living cells (Rodrigo and Jaramillo, 2007) but, as far as we know, this is the first proposal of a multicellular memory device based on a multicellular synthetic design.

The behavior of the device is analyzed in detail with a quantitative mathematical model that incorporates biologically meaningful parameters. The model is used to characterize the feasibility of the flip-flop behavior in a distributed implementation, while also allowing a detailed investigation of the circuit's robustness to parameter changes.

## 2. Materials and methods

### 2.1. Circuit design and mathematical model

**Figure 1:**
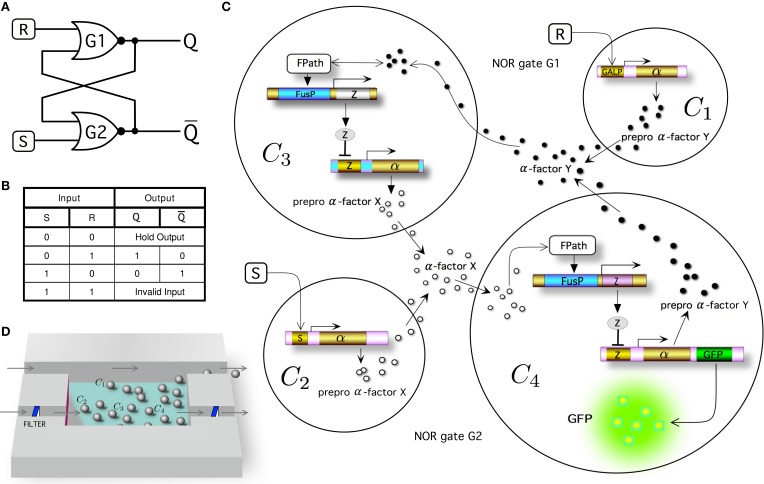
**Multicellular memory circuit**. In **(A)** we display the standard wiring diagram of a set-reset latch or flip-flop circuit, created as a combination of two cross coupled NOR gates. **(B)** Truth table for a NOR gated SR latch. Note that the case where input *R* = *S* = 1 is indicated as an invalid input because it is not allowed. *Q* and *Q* are the two output states. The multicellular consortium is summarized in **(C)**. The designed system is composed of a population of 4 different types of engineered cells, indicated as *C*_1_, *C*_2_, *C*_3_, and *C*_4_ (see Figure S1, for a detailed diagram of the modeled system). Cells *C*_1_ and *C*_2_ respond to inputs *R* (e.g., galactose) and *S* (e.g., doxycyline), respectively. Cells 4 and 3 sense the wiring molecules given by α-factor *X* (e.g., *Saccharomyces*) and α-factor *Y* (e.g., *Candida*), respectively. In both cells *C*_3_ and *C*_4_, a repressor molecule is used to stop the transcription of α-factor, labeled molecule *Z* (e.g., *mLacI*). The selected outputs of the circuit are both prepro α-factors *X* (Cell 3) and *Y* (Cell 4). The readout of the circuit can be monitored in the laboratory by introducing a reporter protein (e.g., green fluorescent protein, GFP, included here in Cell 4). For clarity, the pheromone pathway (FPath) of Cells 3 and 4 is not shown (see Figure 2 and Figure S1, for further details). **(D)** In our model we consider a constant population of cells by means of a constant flux in a microfluidic chamber. The addition of two filters using another chanel can be used to regulate the outflow externally to eliminate the α-factor from the medium without losing cells.

Within electronic design, the most fundamental memory device is the so called SR latch (or SR flip-flop), for which the *S* and *R* inputs stand for set and reset, respectively. This system can be constructed from a pair of cross-coupled NOR logic gates (Figure 1A) implementing the truth table shown in Figure 1B. By adding *S* (i.e., *S* = 1 and *R* = 0), the system moves toward the memory state *Q* = 0 (being *Q* the output and state of the circuit), whereas the system evolves toward the memory state *Q* = 1 by adding *R* (i.e., when *S* = 0 and *R* = 1). In the absence of *S* and *R* (*S* = 0 and *R* = 0), the system remains indefinitely in the previous stable state *Q*. In this kind of circuit, the input combination *S* = 1 and *R* = 1 is not allowed (Santiram, 2002).

Figure 1C summarizes our multicellular implementation, which is intended to serve as a blueprint for a feasible synthetic system. In order to implement this logic architecture in yeast cells, the device is distributed in four different engineered cells, indicated as *C*_1_ − *C*_4_. Cells 1 and 2 (see Figure S1, for further details) are cells that respond to the external inputs, *R* (e.g., galactose) and *S* (e.g., doxycycline), respectively, inducing the secretion of two different α-factor pheromones (α-factor *X* and α-factor *Y*, respectively). These pheromones act as wiring molecules and cells 3 and 4 can sense their external concentrations. Here *C*_3_ has the required receptor (Duntze et al., 1970, 1973) to sense α-factor *Y*, whereas *C*_4_ has a receptor that senses α-factor *X*. Previous studies have demonstrated that it is possible to expresses different receptors in yeast cells, such as receptors from *Saccharomyces cerevisiae* or from *Candida albicans* (Regot et al., 2011). Depending on the specific receptor expressed by the cell, and only in the presence of the corresponding α-factor (α-factor from *Saccharomyces* or α-factor from *Candida*), the pheromone pathway is activated.

Our design makes a significant departure from the standard circuit, that combines NOR gates with feedbacks (Figure 1A). Instead, thanks to the multicellular design, we made use of only 1-input 1-output (NOT and Identity) logic gates (Figure 1C). Specifically, the combination of *C*_1_ − *C*_3_ defines the first NOR gate (G1), whereas the pair *C*_2_ − *C*_4_ implements the second (G2). The activation of the pheromone pathway by the corresponding α-factor induces the expression of protein repressor *Z* (Brent and Ptashne, 1984) which leads to the down-regulation of the expression of the other α-factor, i.e., α-factor *Y* blocks the secretion of α-factor *X* in Cell 3, whereas the presence of α-factor *X* blocks the secretion of α-factor *Y* in Cell 4. In our designed circuit we will use *mLacI* as the repressor molecule (Grilly et al., 2007).

In order to read the state *Q* of the system, a reporter protein (e.g., green fluorescent protein, GFP) is expressed in Cell 4 (or, alternatively, in Cell 3). Figure 1D shows a schematic representation of the microfluidics device that could be used according to our implementation and model assumptions e.g., constant population of cells. The cells are located in a microfluidic chamber from which the population excess is controlled through removal (Bennett and Hasty, 2009). This system is connected to a delivery channel and cells are eventually pushed out from the chamber, flowing to the waste port. The trapping device built with two filters also allows to maintain stable cell populations and tune cell communication by means of a flow rate modifying the concentration of α-factor in medium.

**Figure 2:**
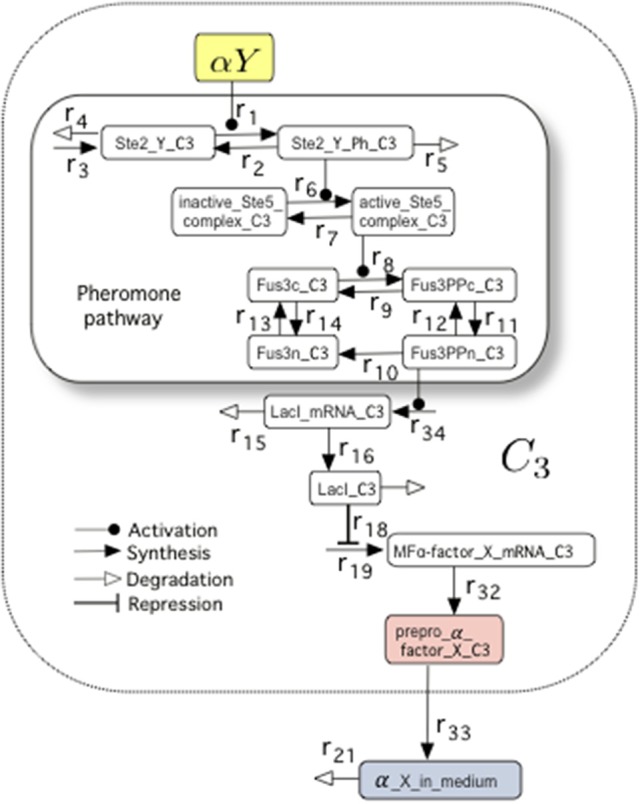
**The molecular composition of the Pheromone Pathway (which appeared encapsulated as FPath in Figure 1C) included in the mathematical model**. Specifically, we display the set of molecular interactions associated to the logic of the NOT cell *C*_3_. In particular, we highlight the pheromone pathway within the smaller box. This model (see Hoffman-Sommer et al., 2012, for further details) allows to incorporate a detailed network of interactions associated to actual signaling pathways of *Saccharomyces* sp. The full diagram is shown in Figure S1 (see also Table S2, for details on the parameters).

To investigate whether our proposed circuit is able to behave as a set-reset flip-flop and how tunable is this behavior, we built a mathematical model, adapted from a previous study by Hoffman-Sommer et al. (2012) who incorporated a detailed description of the actual signaling pathways relevant to our study. Part of this whole circuit is shown in Figure 2. The internal states of Cells 3 and 4, which are responsible for memory maintenance, can be analyzed in detail to monitor the intracellular concentration of the so-called prepro α-factors, which are the precursors of α-factor (Brake et al., 1983). Of note, there is a clear mapping between prepro α-factor levels and reporter protein (GFP) levels, as shown in **Figure 6B**, Figures S2, S6. As we discuss, the GFP can be used to obtain a good readout of the flip-flop signal with the proper GFP degradation rate. Previous research indicates that it is possible to tune the degradation rate of the reporter (Bachmair et al., 1986; Grilly et al., 2007).

A detailed description of the mathematical model, given by a set of ordinary differential equations, is presented in Section S1. The model describes the dynamics and behavior of four types of engineered yeast cells coexisting in a liquid medium, with a culture density of 5 × 10^6^ cells/ml (Hoffman-Sommer et al., 2012). As a first approximation, we consider that the concentrations of different cell types remain constant, i.e., all the cells have the same doubling times. In this sense, it is possible to genetically manipulate a yeast strain in order to avoid cell cycle arrest in the presence of α-factor (Chang and Herskowitz, 1992).

For further details on the mathematical model, parameter values, and initial conditions, we refer the reader to Section 1 in the Supplementary Information and Tables S1, S2.

### 2.2. Numerical tools

Numerical integrations have been carried out using the seventh-eighth order Runge-Kutta-Fehlberg (RKF78) method with automatic step size control and local relative tolerance of 10^−15^.

### 2.3. Measure of the flip-flop performance

**Figure 3:**
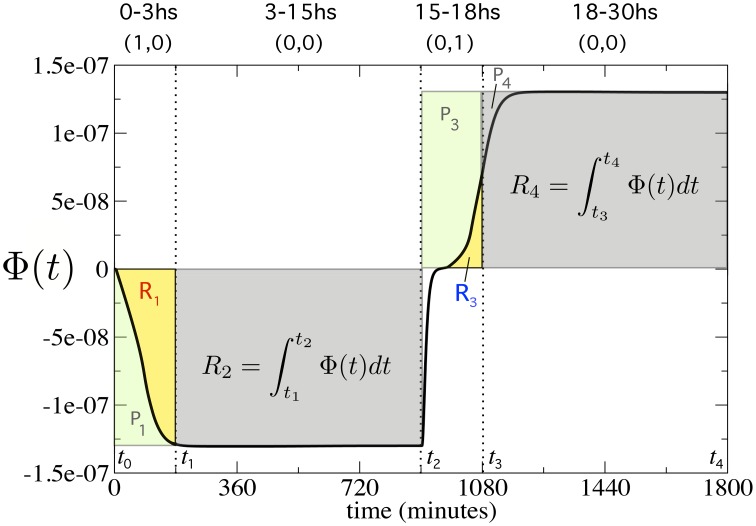
**Measuring the quality of the flip-flop behavior using Φ(***t***) (thick line)**. We computed the perfect areas (filled rectangles) of the first 3 h (*P*_1, 3_) after inputs (1, 0) and (0, 1), and the areas (*P*_2, 4_) 12 h after inputs (0, 0). Then, we computed the real areas from Φ(*t*) for four different time periods (yellow and gray regions), denoted as *R*_1, .., 4_. These four time periods are fixed at *t*_0_ = 0, *t*_1_ = 180, *t*_2_ = 900, *t*_3_ = 1080, and *t*_4_ = 1800 min, respectively (see Model and Methods Section for further details).

In order to properly measure the accuracy of the flip-flop, it is crucial to define a quality parameter incorporating both the time and amplitude responses. The time response refers to the time required for the transition from one memory state to another when external inputs *R* and *S* are changed. On the other hand, the amplitude response refers to the separation between the 0 and 1 logic levels. For a given set of parameters Ω = {ω_1_, ω_2_, …}, which we simply indicate as ω_*i*_, the behavior of the circuit can be described in terms of the difference between the concentrations of the prepro α-factors in Cells 3 and 4. This difference is described by Φ(ω_*i*_, *t*), defined as

(1) $$\begin{array}{l} \Phi \left(\omega_{i},t\right)=\phi_{x}\left(\omega_{i},t\right)-\phi_{y}\left(\omega_{i},t\right), \end{array}$$

where ϕ_*x*_(ω_*i*_, *t*) and ϕ_*y*_(ω_*i*_, *t*) are the concentrations of prepro α-factors *X* and *Y* presents in Cell 3 and Cell 4. For the sake of simplicity, hereafter Φ(ω_*i*_, *t*) will be named Φ(*t*). In a nutshell, we need to compare the predicted, ideal behavior of a flip-flop (rectangular areas, *P*_1_…*P*_4_ in Figure 3) with the actual changes associated to a continuous implementation as given by the response Φ(*t*), which defines different areas (thick line, *R*_1_…*R*_4_, in Figure 3).

In order to measure quality, we need to consider: (i) the time response upon the specific external input combinations *R* = 1, *S* = 0 and *R* = 0, *S* = 1, which write 1-bit information to the memory device; and (ii) the maintenance of the memory when *R* = 0 and *S* = 0. The ratio between the real areas *R*1 and *R*3 and the perfect areas *P*1 and *P*3 determine the goodness of the time response, named *S*_*r*_.

Similarly, the ratio between the real areas *R*2 and *R*4 and the perfect areas *P*2 and *P*4 define the goodness of the persistent memory, labeled *S*_*m*_. These terms can be calculated according to

$$\begin{array}{l} S_{r}=\frac{1}{2}\left(\frac{R_{1}}{P_{1}}+\frac{R_{3}}{P_{3}}\right),\text{ and }S_{m}=\frac{1}{2}\left(\frac{R_{2}}{P_{2}}+\frac{R_{4}}{P_{4}}\right), \end{array}$$

where *R*_*i* = 1, …4_ and *P*_*i* = 1, …, 4_ denote the real and the perfect areas, respectively, which are computed as follows. Each of the real areas *R*_*i*_ is given by the integral of Φ(*t*) in the four different time intervals corresponding to the changes in inputs. The perfect areas *P*_*i*_ are delimited by the maximum and minimum values of Φ(*t*). Then, *S*_*r*_ and *S*_*m*_ are given by

$$\begin{array}{l} S_{r}=\frac{1}{2}\left(\frac{1}{P_{1}}\underset{R_{1}}{\underset{︸}{\int_{t_{0}}^{t_{1}}\theta_{1}\left(t\right)\cdot \Phi \left(t\right)dt}}+\frac{1}{P_{3}}\underset{R_{3}}{\underset{︸}{\int_{t_{2}}^{t_{3}}\theta_{3}\left(t\right)\cdot \Phi \left(t\right)dt}}\right), \end{array}$$

where we define the functions:

$$\theta_{1}(t)=\{\begin{array}{ll} 1 & \text{if }\Phi (t)<0,\\ 0 & \text{otherwise}, \end{array}\;\theta_{3}(t)=\{\begin{array}{ll} 1 & \text{if }\Phi (t)>0,\\ 0 & \text{otherwise.} \end{array}\;$$

For the memory term, we have

$$\begin{array}{l} S_{m}=\frac{1}{2}\left(\frac{1}{P_{2}}\underset{R_{2}}{\underset{︸}{\int_{t_{1}}^{t_{2}}\Phi \left(t\right)dt}}+\frac{1}{P_{4}}\underset{R_{4}}{\underset{︸}{\int_{t_{3}}^{t_{4}}\Phi \left(t\right)dt}}\right). \end{array}$$

We used the following period times in our simulations: *t*_0_ = 0 min, *t*_1_ = 180 min, *t*_2_ = 900 min, *t*_3_ = 1080 min, and *t*_4_ = 1800 min (see Figure 3), if not otherwise specified. Notice that by integrating on both the negative and positive values of Φ(*t*) in the memory states, we ensure that the quality measure can also work in asymmetric circuits (that are analyzed in Section S-III). In fact, when introducing asymmetries between cells, Φ(*t*) could change sign in the memory states. For instance, in Figure S6(b.2), the value of Φ(*t*) changes from negative to positive in the first memory state. Our approach allows this behavior to be penalized, since the positive area in this time period will be subtracted from the one we computed.

We can define a quality factor Θ_*S*_, which refers to the shape of the time response, according to a Pareto function

(2) $$\begin{array}{l} \Theta_{S}=\lambda \cdot S_{r}+\left(1-\lambda \right)\cdot S_{m}, \end{array}$$

For those cases where the minimal conditions of a proper flip-flop behavior are not found, we will assume Θ_*S*_ = 0. More specifically, we will assume zero quality if no negative values of Φ(*t*) are found during the first 15 h. Similarly, we will assume zero quality if Φ(*t*) has no positive values during the last 15 h (assuming the inputs series: (1, 0) at 3 h; (0, 0) at 12 h; (0, 1) at 3 h; and (0, 0) at 12 h). The parameter λ ∈ [0, 1] weights the contribution of each component *S*_*r*_ and *S*_*m*_. If λ = 1, Θ_*S*_ will only account for the goodness of the circuit response during activation while λ = 0 only considers memory persistence, i.e., the dynamics in the absence of external inputs. The Θ_*S*_ values range from 0 to 1. It is worth noting that in the case of an ideal flip-flop signal, Θ_*S*_ = 1 for all values of λ, since the system would have a perfect response to all inputs and a perfect memory, thus behaving like a sharp, perfect digital signal.

However, a proper digital behavior should not be enough if the signal amplitude, i.e., the difference between low and high logic levels, is not large enough to be detected in a real experimental setup. The term Θ_*A*_(ω_*i*_), hereafter named Θ_*A*_ for simplicity, accounts for this effect. Θ_*A*_ can be defined as

(3) $$\begin{array}{l} \Theta_{A}=\Theta_{S}\cdot \frac{m\left(\omega_{i}\right)\cdot M\left(\omega_{i}\right)}{m_{T}\left(\Omega \right)\cdot M_{T}\left(\Omega \right)}, \end{array}$$

where *m*(ω_*i*_) and *M*(ω_*i*_) denote the minimum and maximum values of Φ(*t*), respectively. Since *m*(ω_*i*_) and *M*(ω_*i*_) are parameter-dependent, a normalization factor *m*_*T*_(Ω) · *M*_*T*_(Ω) is used. *m*_*T*_(Ω) and *M*_*T*_(Ω) indicate, respectively, the largest *m*(ω_*i*_) and *M*(ω_*i*_) values in Ω, with *m*_*T*_(Ω) = min{*m*(ω_*i*_), *m*(ω_2_), …}, and *M*_*T*_(Ω) = max{*m*(ω_*i*_), *m*(ω_2_), …}.

## 3. Results

All of the results presented in the following sections have been obtained by numerically solving the model equations presented in Section S1.

### 3.1. Circuit dynamics and flip-flop behavior

One of the advantages of our multicellular implementation is the possibility of manipulating several parameters, such as input concentrations or inflow-outflow rates, that could affect the circuit response without additional genetic engineering. This allows the circuits response to be optimized just by tuning the experimental setup. As our first approach, we investigate the behavior of the designed circuit by using biologically meaningful parameters values from the literature (Tables S1, S2), considering that the cell population remains constant (see Section S1).

We explored the dependence of this system with respect to the dilution rate of α-factors in medium (*k_α_X, Y_deg_*). This includes both the spontaneous decay of the molecules and their clearance from the medium due to the presence of a constant and regulable outflow, which can be easily tuned for an optimal response in a microfluidic environment. In all of our analyses we allowed each cell type to reach equilibrium independently (i.e., running the system for 7 h without cell communication) before introducing the first activation input (1, 0), and then setting the time to *t* = 0. Numerical simulations revealed that, in a given and wide region of the parameter space, the circuit behaves as a flip-flop, responding correctly to the activation inputs (1, 0) and (0, 1) and being able to maintain the state upon the memory inputs (0, 0). The goodness of the flip-flop behavior was computed using the two complementary quality measures, given by Θ_*S*_ and by Θ_*A*_ (see Equations 2 and 3).

**Figure 4:**
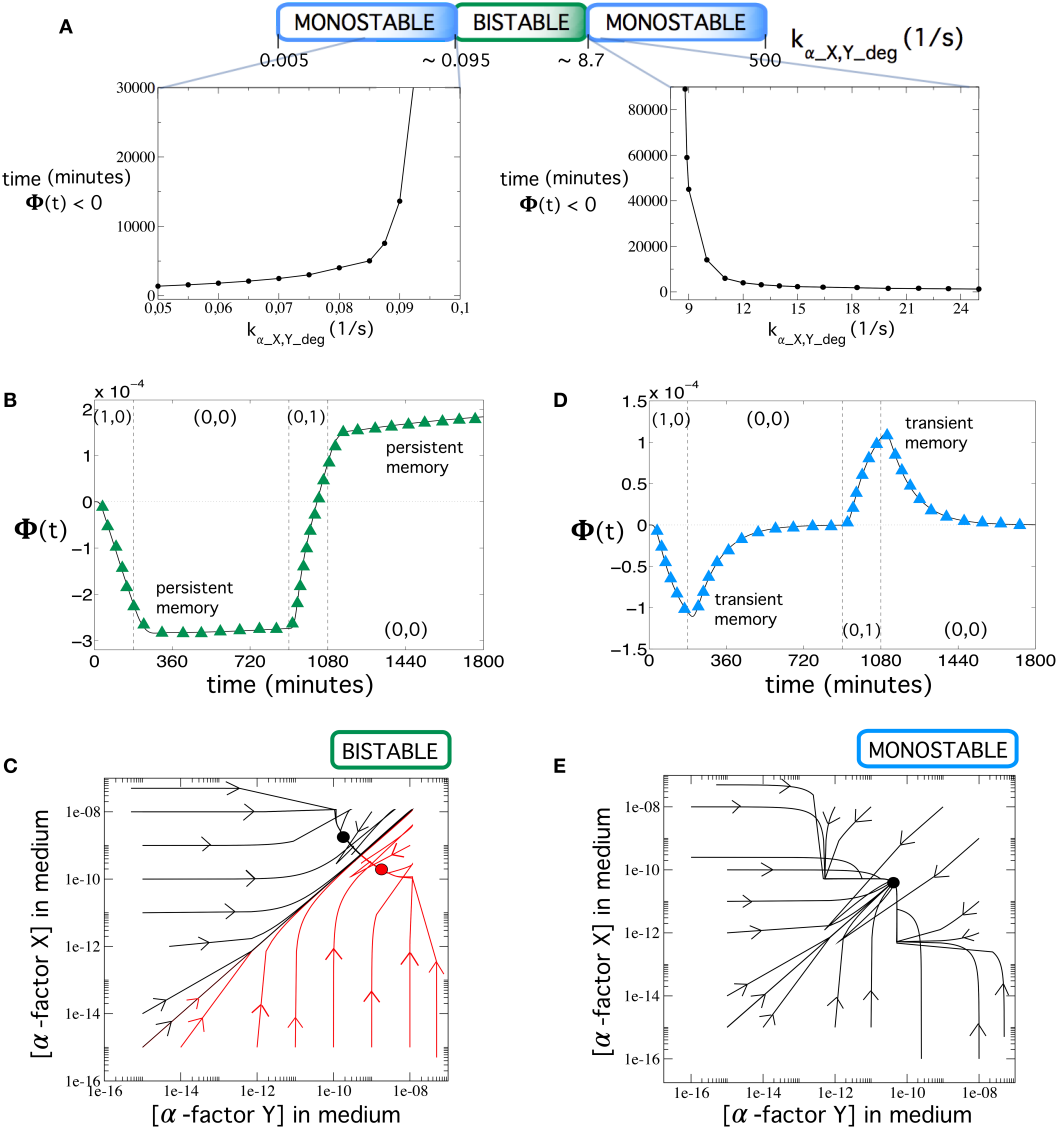
**(A)** Stability changes at increasing the values of the elimination rate of α-factor in medium, *k_α_X, Y_deg_*. Note that the values of *k_α_X, Y_deg_* in the top panel are not scaled. Below, we show the time that Φ(*t*) remains negative (memory time) during the second input (0, 0) after introducing input (1, 0) for 3 h. We specifically show two ranges for the monostable states. Notice that both times greatly increase as the bistable scenario is approached. **(B)** Dynamics of Φ(*t*) in the bistability regime using [*input*] = 40 μg∕ml and *k_α_X,Y_deg_* = 0.134 s^−1^. **(C)** Phase portrait displaying bistability using the same parameter values as in **(B)**. Here, the circuit dynamics can achieve two different equilibrium points corresponding to the dominance of α-factor *Y* in medium (green circle) and the dominance of α-factor *X* in medium (black circle). **(D)** Dynamics of Φ(*t*) in the monostability regime with [*input*] = 192 μg∕ml and *k_α_X,Y_deg_* = 30 s^−1^. Notice here that memory is transient (see also **Figure 7D**; green trajectories) and **Figure 8E** (green and blue trajectories) for examples of long transient memories). **(E)** Phase portrait displaying monostability using the same parameter values as in **(D)**. Each plot shows several trajectories starting from different initial conditions of both α-factors in medium (represented in log-log scale). Each trajectory in the phase portraits was obtained from 300-h simulations using the values from Table S2. We notice that in the phase portraits, the concentrations of both α-factors in medium were typically very low, but there is a difference of about one order of magnitude between the stable equilibria in the bistability regime. The arrows indicate the direction of the flow.

As the first result, we observe a clear dependence on the device's behavior with respect to the elimination rate. It is well-known that flip-flops are bistable systems (Cherry and Adler, 2000; Santiram, 2002). A bistable system may reach one of the two possible stable states, depending on the initial conditions, and remain there unless a new input forces the state to change (persistent memory). On the other hand, a monostable system does not allow for permanent memory. However, monostable devices can remain at a given unstable state for a long period of time. This type of systems can be useful for applications demanding transient, rather than permanent memories. Interestingly, our multicellular device is able to exhibit both dynamics, i.e., bistability and monostabiliy, as shown in Figure 4, where we plot several trajectories in the phase space using different initial conditions.

Our simulations show that, within the ranges 0.005 ≤ *k_α_X, Y_deg_* ≲ 0.095 and 8.7 ≲ *k_α_X, Y_deg_* ≤ 500, the circuit is monostable and displays transient memory, while for a wide range of elimination rates (0.095 ≲ *k_α_X, Y_deg_* ≲ 8.7), the system can undergo bistability (Figure 4A). In Figure 4A, we also display the times when Φ(*t*) remained negative in the monostable ranges (as a way to compute the transient duration of the memory). These times are finite in the monostable regimes since Φ(*t*) → 0 for *t* → ∞: at equilibrium, both outputs of the circuit, i.e., prepro α-factors *X* and *Y*, equal their values because of the symmetry of the circuit (notice that the equilibrium point in Figure 4E is placed in the diagonal). To compute these times, we ran simulations for each value *k_α_X, Y_deg_*, computing the time when Φ(*t*) was negative during the second input (0, 0), after introducing input (1, 0) for 3 h. The results show that the closer the elimination rate of α-factor is to the bistability regimes, the longer the times.

The time-course evolution of Φ(*t*) is shown in Figures 4B,D, respectively. In the example shown in Figure 4B, using *k_α_X,Y_deg_* = 0.134 s^−1^, the system reaches the stable state after input (1, 0) and stays in that state [during input (0, 0)] until the second input (0, 1) is applied, thus keeping persistent memory. Figure 4D shows an example (now with *k_α_X,Y_deg_* = 30 s^−1^) where the system is pushed toward an unstable state (Φ(*t*) < 0) upon external inputs (1, 0). The system slowly starts to move toward the stable state before changing to inputs (0, 1), thus exhibiting transient memory. Figures 4C,E show the trajectories corresponding to both the bistable and monostable circuits. From the previous results, we can conclude that the same device can be used to implement permanent memories (bistability) or transient memories (monostability) by only tuning the elimination rate of the α-factors in the medium.

**Figure 5:**
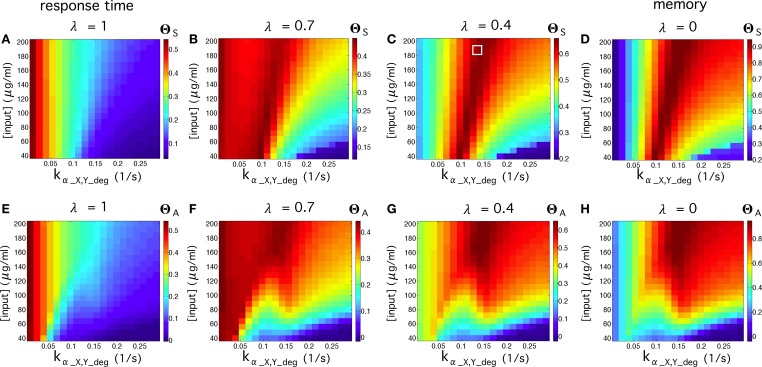
**Flip-flop qualities, Θ_***S***_, for different values of input concentrations and elimination rates of α-factor in medium ***k_α_X, Y_deg_***, using the parameter values from Table S2**. In the first row **(A–D)**, we display the quality values without considering the amplitude, Θ_*S*_, while in the second row **(E–H)** we measured the quality considering the amplitude, Θ_*A*_. In each row, we display four cases using different values of λ (see Equation 2 in the main manuscript). The cases for λ = 1 denote the regions of the parameter space analyzed in which the circuit responds better to activation inputs. The cases with λ = 0 display the parameter values that give place to longer memories. We also display intermediate values of λ. The white square inside **(C)** indicates input and elimination rates values of the α-factor that will be used in most of our simulations (if not otherwise specified), since such values give place to good signal quality.

In order to test the quality of our multicellular flip-flop circuit, we have explored the quality response upon changes in two externally controllable magnitudes: the input concentrations and elimination rates of α-factors in medium. The results are shown in Figure 5. First, we have explored the quality of the flip-flop response with respect to the time responses, i.e., how fast and permanent the changes in the device states are in response to the external inputs *R* and *S*, quantified by Θ_*S*_. Looking only to time response, i.e., λ = 1 (Figure 5A), we observe that lower elimination rates provides faster responses. Moreover, by focusing only on optimal memory persistence, i.e., λ = 0 (Figure 5D), this can be achieved by tuning the elimination rates to optimal values in the range 0.1 ≤ *k_α_X, Y_deg_* ≤ 0.17). Lower elimination rates lead to a poorer behavior because the persistence of the wiring molecules in the medium prevents state changes. Similarly, higher values lead to excessive removal of the wiring signals in the system. The optimal region is not very dependent on the specific input concentrations.

Higher impact is observed in the region of higher elimination rates, where an increase of the input concentrations leads to improved flip-flop quality. Considering the contribution of the signal amplitude as an additional element in the quality assessment, described by Θ_*A*_, we observe (Figures 5E–H) that the dependence on input concentrations becomes stronger. Here, the optimal flip-flop region remains in a similar range of elimination rates, but higher input concentrations significantly improve the quality of the flip-flop. In summary, our results indicate that whereas time response dynamics are basically dependent on the elimination rates, the amplitude of the response depends on the concentration of the external inputs *R* and *S* responsible for inducing α-factor secretion in Cells 1 and 2. This decoupling between input concentrations and elimination rates provides a very flexible way to optimize the experimental device.

**Figure 6:**
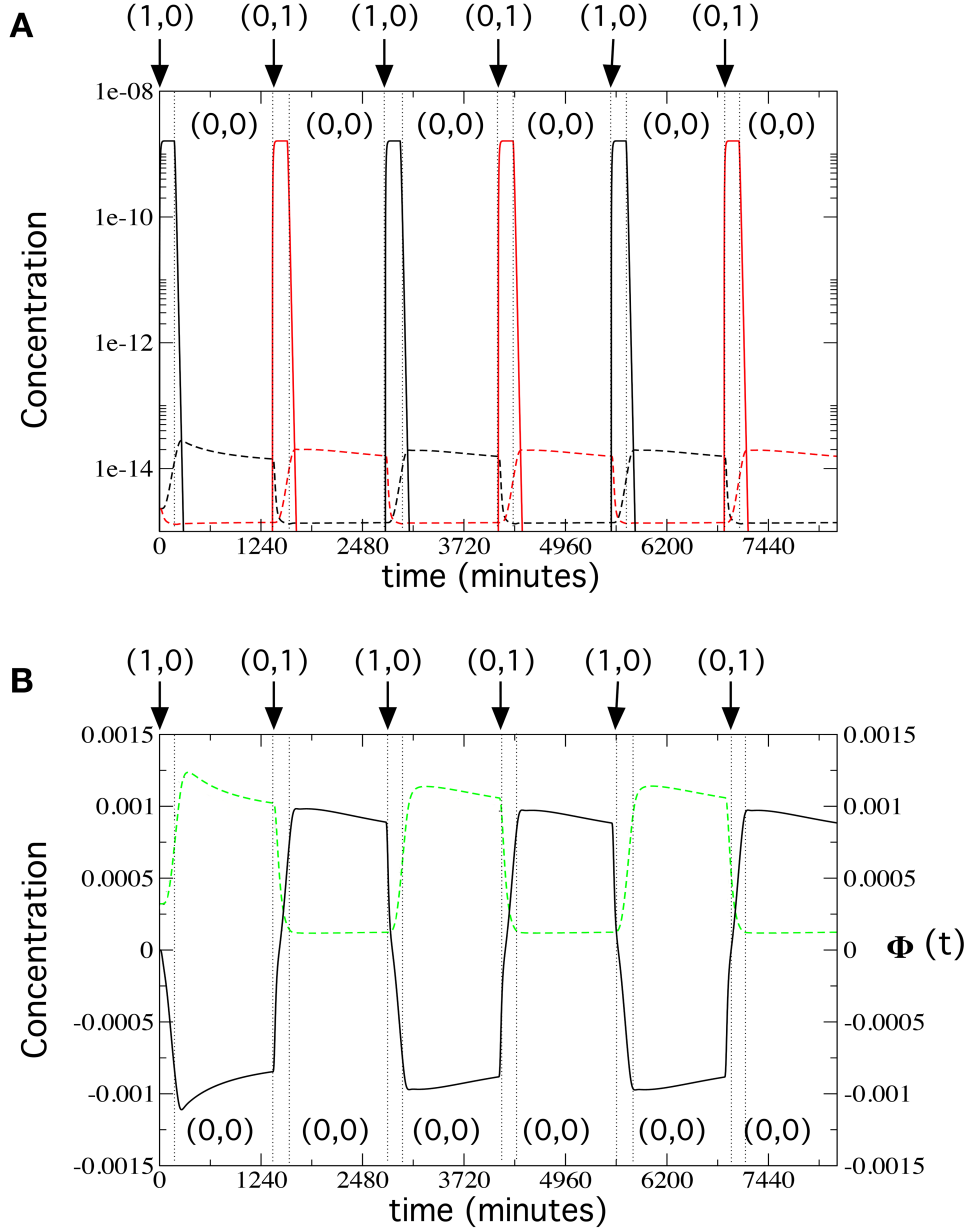
**Long-term flip-flop behavior in the bistability regime, using the parameter values from Table S2, with ***k_GFP_deg_*** = 0.00215 s^−1^, [***input***] = 192 μg/ml, and ***k_α_X,Y_deg_*** = 0.134 s^−1^**. We simulated the system for 138 h, repeating the following sequence of activation input and memory inputs three times: (1, 0): 3 h; (0, 0): 20 h; (0, 1): 3 h; and (0, 0): 20 h (input changes are indicated with vertical dotted lines). **(A)** Release dynamics of α-factor by Cells 1, 2 (solid lines) and Cells 3 and 4 (dashed lines) cells (in linear-log scale). We display the α-factor Y in medium (black) released by Cells 1 and 4, and the α-factor *X* in medium (red), released by cells 2 and 3. **(B)** Time dynamics of Φ(*t*) (solid black line) and GFP (green dashed line), with the GFP gene inserted next to the α-factor Y gene in Cell 4.

We can now address the long-term dynamics of our flip-flop circuit (Figure 6). We ran a 138-h simulation, in which inputs follow the usual sequence, i.e., activation inputs (1, 0) and (0, 1) for 3 h and memory inputs (0, 0) for 20 h. Here, we used the values from Table S2 with input concentrations of 192 1.5 mm μg/ml. In Figure 6A, we plot the contribution of the α-factors in medium secreted by each of the four types of cells in the circuit. Cells 1 and 2 behave as on-off switches, as expected, with a very large production of α-factor during the activation inputs, thus triggering the response of Cells 3 and 4 that is maintained during periods with (0, 0) inputs. In Figure 6B, we plot, for the same simulation, prepro α-factor and GFP concentrations for Cell 4. These time series reveal that both the changes in inputs as well as the memory lengths are maintained over time; therefore, the long-term flip-flop dynamics are stable.

### 3.2. Molecular parameter exploration

Beyond the tunable response associated to external inputs, we can also consider changing the internal circuit architecture, with minimal engineering by modifying the Cells 3 and 4. The fundamental control points in this architecture are (i) the transcription rate of the prepro α-factors mRNAs (γ); (ii) the transcription rate of the mRNA for repressor protein *Z* (*k*_34_); and (iii) the repression efficiency (β_*c*_). In order to increase the transcription rate of *Z* and the prepro α-factor, several copies of the same gene can be introduced in the cell. Alternatively, promoter changes can reduce the transcription ratio. Repressor efficiency of *Z* can also be changed by adding more than one repressor-binding domain to the *Z*-promoter. We computed the quality of the flip-flop after tuning these key parameters. The studied ranges are presented in the caption of Table S2. The equations to compute the quality of the flip-flop [see Equations (2)–(3)] include the constant λ, which determines the weight that we give to the response amplitude or memory time of the circuit. In most of the next analyses, we will fix λ = 0.4. However, in the next section we will analyse the effect of every parameter on the response amplitude and memory times for different values of λ.

**Figure 7:**
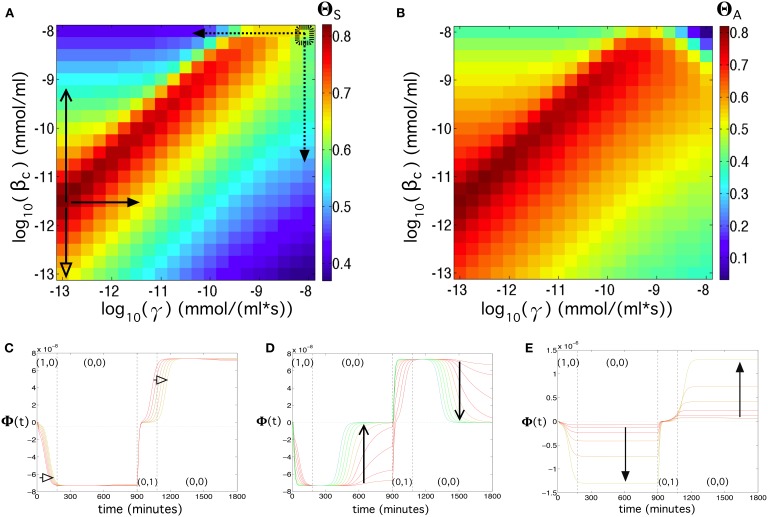
**Flip-flop behavior changing the transcription rate of α-factor (γ) in the Cells 3 and 4, and the repression constant of LacI (β_***c***_), using [***input***] = 192 μg/ml, ***k_α_X,Y_deg_*** = 0.134 s^−1^, and λ = 0.4 (see Equation 2 in the main manuscript)**. **(A)** Quality of the flip-flop, Θ_*S*_ (see Model and Methods in the main manuscript), in the parameter space (γ, β_*c*_). The thick dashed rectangle displays the values of γ and β_*c*_ obtained from the literature (see Table S2). **(B)** Same as in **(A)** but now considering the amplitude of the flip-flop, computed from Θ_*A*_. Below we show three panels containing time series using parameter values indicated with the solid arrows in **(A)**. For **(C)** we set γ = 10^−12.75^ mmol∕(ml · s) and 10^−13^ ≤ β_*c*_ ≤ 10^−11.5^ mmol/ml. For (d) we set γ = 10^−12.75^ mmol∕(ml · s) and 10^−11.5^ ≤ β_*c*_ ≤ 10^−9.15^ mmol/ml. In **(E)** we used β_*c*_ ≤ 10^−11.5^ mmol/ml, and 10^−12.75^ ≤ γ ≤ 10^−11.25^ mmol∕(ml · s). The colors of the time series correspond to the color scale of the flip-flop qualities displayed in **(A)**. The dashed arrows in **(A)** indicate parameter ranges used to compute the response and memory times of the flip-flop (see Figures S4E,F, S5A,B).

By tuning γ and β_*c*_ we found that, for a very wide region of this parameter space (note that the axes are in log-log scale), the circuit behaves well. The best qualities are found at decreasing values of γ and β_*c*_, i.e., γ ~ 10^−13^ mmol∕(ml · s) and β_*c*_ ~ 10^−11.75^. These values do not correspond to the values shown in Table S2 (indicated with a small dashed square overlapping the parameter space in Figure 7A). This change in the values γ and β_*c*_ could be obtained, for instance, by decreasing the mRNA production of the two α-factors (decrease in γ) or by inducing a higher repression of Z (decrease in β_*c*_ value). The comparison between these two optimal parameter values with the values found in Table S2 reveal that, while we might obtain a flip-flop quality of Θ_*S*_ = 0.6 in the available data, such a quality could be augmented up to Θ_*S*_ = 0.82 by decreasing such parameters. The values from Table S2 reveal that the flip-flop under this parameter combination would have a low quality in terms of amplitude (Θ_*A*_ = 0.2). However, this can be counterbalanced by a slight decrease of γ and/or β_*c*_ that may enhance the amplitude, especially γ, which has grater influence on the signal's amplitude (see Figures 7B–E).

**Figure 8:**
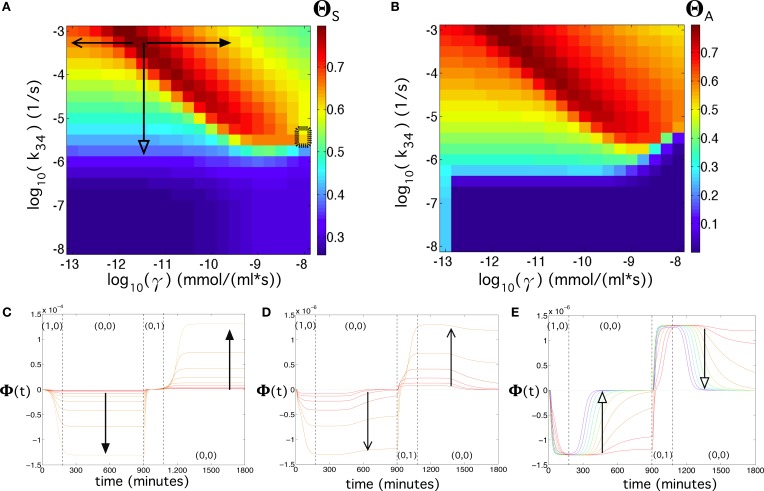
**Flip-flop behavior at changing values of the transcription rate of α-factor, γ, and of the Fus3ppn-activated transcription of the LacI mRNA, ***k***_34_, using [***input***] = 192 μg/ml, ***k_α_X,Y_deg_*** = 0.134 s^−1^, and λ = 0.4 (see Equation 2 in the main manuscript)**. **(A)** Quality of the flip-flop, Θ_*S*_, in the parameter space (γ, *k*_34_). Here, we also display, with a thick dashed rectangle, the corresponding values of γ and *k*_34_ obtained from the literature (Table S2). **(B)** Same as in **(A)** but using the quality measure including the amplitude of the flip-flop, Θ_*A*_. Below we show three panels containing time series using different parameter values indicated with the arrows in **(A)**. In **(C)** we set *k*_34_ = 10^−3.25^ s^−1^ and 10^−11.5^ ≤ γ ≤ 10^−9.5^ mmol∕(ml · s). In **(D)** we also used *k*_34_ = 10^−3.25^ s^−1^ and 10^−12.75^ ≤ γ ≤ 10^−11.5^ mmol∕(ml · s). In **(E)** we set γ = 10^−11.5^ mmol∕(ml · s) and 10^−5.75^ ≤ *k*_34_ ≤ 10^−3.25^ s^−1^. Here, the colors of the time series also correspond to the color scale of the flip-flop quality, Θ_*S*_, displayed in **(A)**. The dashed arrow in **(A)** indicates the parameter range for *k*_34_ used to compute the response and memory times time to state after input introduction and the memory of the flip-flop (see Figures S4E,F in the main manuscript and Figures S5C,D).

**Figure 9:**
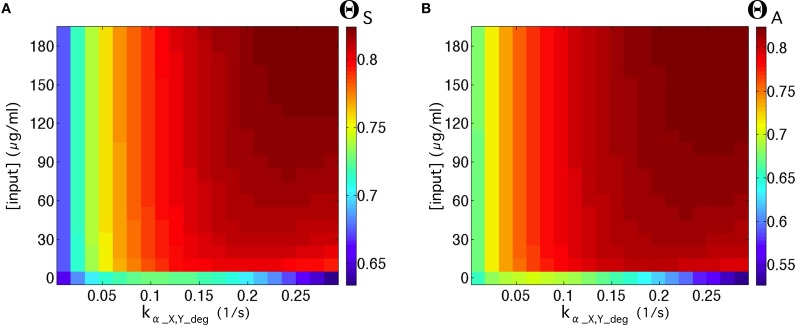
**(A)** Quality of the flip-flop, Θ_*S*_, in the parameter space ([*input*], *k_α_X, Y_deg_*), with λ = 0.4 (see Equation 2 in the main manuscript) using the values of Table S2, except for γ = 10^−13^ mmol∕(ml · s) and β_*c*_ = 10^−11.75^ mmol/ml. In **(B)** we show the same as in **(A)** but considering the amplitude of the signal, Θ_*A*_. In both panels we used the inputs series (1, 0): 3 h; (0, 0): 12 h; (0, 1): 3 h; and (0, 0): 12 h (compare with Figures 4C,G).

Exploring (γ, *k*_34_) also reveals a wide, high-performance region (Figure 8). Here, we notice that the values for γ and *k*_34_ presented in Table S2 already provide a high flip-flop quality (Θ_*S*_ = 0.63 for λ = 0.4), although with low amplitude. Again, a slight modification of these parameters, e.g., a slight increase of both γ and *k*_34_, may cause an increase and an enhancement of the signal (see Figure 8B). Figures 8C,D also reveals that parameter γ, compared to β_*c*_ and to *k*_34_ has a strong effect on the amplitude of the signal. Figure 8E displays the effect of decreasing parameter *k*_34_, which involves a decrease of the flip-flop signal and a transition from persistent to transient memory. The flip-flop devices can be optimized by introducing genetic modifications as key points of the architecture. We repeated the analyses shown in Figure 5 for λ = 0.4 using γ ~ 10^−13^ mmol∕(ml · s) and β_*c*_ ~ 10^−11.75^ mmol/ml, which were previously identified as the parameter values that enhanced the flip-flop quality. The results can be seen in Figure 9 (see also Section SII).

### 3.3. Parameter exploration by tuning λ: Response time vs. memory

In an ideal memory device, the quality measure Θ_*S*_ should be 1 independently of the specific value of the parameter λ. This represents an optimal behavior both in time responses and in memory persistence. In this section, we explore which parameters are critical to obtain this type of behavior. As we previously commented on, depending on the value of λ, the quality measure for a given set of parameter values will give a different weight to the time response or to the memory persistence. By doing so, we will explore the effects of varying λ on the quality of the signal, identifying those parameters regions where the circuit has a better response or a longer memory. First, we explored the impact of the externally tunable parameters, i.e., we analyzed the quality of the system by changing the input concentration and the elimination rate of the α-factors for different values of λ.

**Figure 10:**
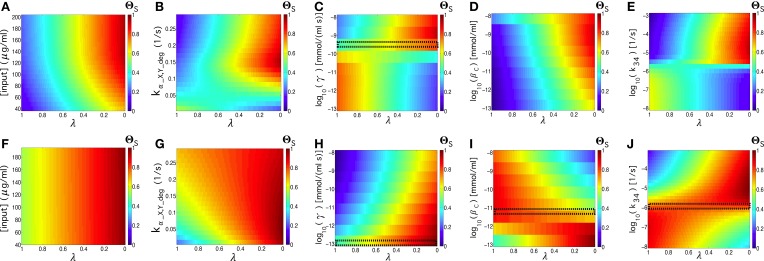
**Quality values, Θ_***S***_, of the circuit's signal, tuning λ thus considering the full range between the response time (λ = 1) and the memory (λ = 0) of the circuit**. We tune the following parameters: [*input*], *k_α_X, Y_deg_*, γ, β_*c*_, and *k*_34_, see Table S2. In **(A–E)**, we use the values from Table S2, while in **(F–J)** we repeated the same analysis using parameter values that lead to better flip-flop quality, with β_*c*_ = 10^−11.75^ mmol/ml and γ = 10^−13^ mmol∕(ml · s). In all of the analyses, we set [*input*] = 192 μg∕ml, and *k_α_X,Y_deg_* = 0.134 s^−1^. The small black rectangles display the parameter values that produce good flip-flop qualities independently of λ. For the sake of comparison, the values of Θ_*S*_ in all of the parameter spaces have been normalized.

Figures 10A–E corresponds to the parameter values shown in Table S2, except for the tunable parameter. Similarly, in the images on the lower part (Figures 10F–J), we use γ ~ 10^−13^ mmol∕(ml · s) and β_*c*_ ~ 10^−11.75^, which provide an optimal flip-flop signal. The input concentrations and elimination rate are fixed to [*input*] = 192 μg∕ml and *k_α_X,Y_deg_* = 0.134 s^−1^, except for the figures where we use them as the tunable parameter. We can observe that parameters such as γ, β_*c*_, and *k*_34_ allow for good quality independent of the λ values (see the small, dashed rectangles in Figures 10C,H–J), indicating that this device configuration provides both good quality in time responses and in memory persistence.

Focussing on the externally controllable parameters, i.e., input concentration and elimination rates, we observed different effects. Whereas modifying the input concentrations had no significant impact on the dependence of quality factor with respect to λ, the elimination rate, in some cases (Figure 10G), makes the quality factor less dependent on when the elimination rate increases, indicating a significant improvement on the time responses while maintaining the quality of the memory persistence. Similar results have been obtained using asymmetric scenarios (see Section S-III and Figure S6 in the Supplementary Information).

## 4. Discussion

Synthetic memory devices have been implemented in the last decade (Burrill and Silver, 2010; Inniss and Silver, 2013). A landmark paper (Gardner et al., 2000) first established the basis for molecular memory with the toggle switch in *E. coli*, based on a bistable design with cross-repression. A different architecture was implemented in yeast (Ajo-Franklin et al., 2007), where transcriptional positive feedback with sensitivity to cell growth was used. Here, memory was sustained by an autoregulatory transcriptional positive feedback. Other devices such as conditional memory systems (Fritz et al., 2007) have been explored. Other implementations based on the expression of specific recombinases in bacteria (Siuti et al., 2013) have been also developed. Switchable memories have been built using DNA biochemistry *in vitro* (Padirac et al., 2012; Inniss and Silver, 2013).

In this work, we have computationally explored the feasibility of implementing memory circuits using a multicellular embodiment, considering engineered yeast cells as our case study. By splitting different parts of the flip-flop circuitry among different engineered cells, a simple, non-standard design is obtained where memory is specifically located in two cell types. This is actually an interesting feature of our multicellular design, which spatially separates memory components in well defined modules, somewhat resembling standard circuit designs. Using realistic parameter settings and an appropriate measure of circuit performance, we have shown that reliable memory can be achieved. Indeed, the exploration of the parameter space have revealed that the flip-flop behavior is very robust to parameter changes. The evolutionary robustness of circuits' responses have been recently discussed in Sleight et al. (2010). These authors suggested that inducible promoters turn circuits more stable from an evolutionary point of view. Since our circuit contains such promoters, one might expect that such circuit may continue working under long evolutionary times.

As defined in our implementation, it has been shown that the circuit could be easily tuned in a microfluidic environment by controlling flow rates and input concentrations. While a constant population can be maintained with a controlled outflow, the elimination of α-factor in medium can be manipulated by the second outflow along the chambers with the filters (see Figure 1D). This allows the device behavior to be optimized or even switched from bistable to monostable dynamics without the necessity to largely modify the architecture of the designed synthetic circuits. Both permanent and transient memories can be achieved by tuning these external parameters.

Moreover, further architectural modifications affecting the internal molecular parameters of the designed circuit could be done to enhance the flip-flop signal. For instance, Cells 1 and 2 transcribe α-factor upon input addition, thus, the rate of α-factor transcription depends on which promoter is triguered by the input stimuli. Still, there is room for tuning the response by addition of copies of the plasmid or engineering the number of copies of α-factor of each construct. Concerning Cells 3 and 4, the pheromone transcriptional response (the transcription of LacI in response of α-factor) can be tuned by choosing any of the promoters that are up-regulated by α-factor presence. A list of those yeast promoters and their strength can be found in Christopher et al. (2000). To tune the strength of the repression can be reduced by increasing amounts of IPTG (Grilly et al., 2007). Also the mlacI repressor can be changed for the TetA repressor or the natural yeast repressors as sn66 or Tup1 (Bellí et al., 1998). Also, more operons can be aded upstream of the repressible promoter. Since the constitutive-repressible promoter that controls α-factor is engineered by upstream addition of the repressor binding sites (Cox et al., 2007), the constitutive transcription of α-factor can be increased or decreased by changing the strength of the constitutive engineered promoter. A good set to check the strength of a constitutive promoter can be found in the data set of Huber et al. (2006).

As previously mentioned, previous studies on synthetic memory systems have focused on single-cell implementation requiring a logical scheme that is close to the standard one (Rodrigo and Jaramillo, 2007). The presence of memory is also crucial in other types of more complex designs (Lu et al., 2009) including associative learning (Fernando et al., 2009; Sorek et al., 2013) but little has been done so far to explore multicellular memories, despite the potential for flexible designs, reusability and limited engineering. Our approach could be particularly useful in the context of systems -such as the microbiome- where many interacting microbial strains are present. If we imagine the microbiome as a large, living biological computer, our consortia could act as small added circuits capable of locally modifying undesirable behaviors.

Future work should explore the applicability of multicellular consortia to develop more complex synthetic constructs with memory beyond 1-bit. For instance, a device capable of remembering a biological experience might be utilized in the long-term study of particular cells with a heterogeneous population following a defined event, or applied in industry for the sustained production of desired substances, e.g., proteins, after input induction. We believe that the modular architecture of our multicellular design allows to easily scale up our current design to these more general purposes.

## Author contributions

JM and RS designed the multicellular circuit. JS, AB, JM, and NC performed the numerical simulations and analyzed the data. All authors built the mathematical model and wrote the article. JS and AB contributed equally to this paper.

## Funding

This work was partially funded by the European Research Council Grant ERC SYNCOM 294294 (JM, RS, AB, NC), by grants of the Botin Foundation, by Banco Santander through its Santander Universities Global Division (RS, JS), and the Santa Fe Institute (RS).

### Conflict of interest statement

The authors declare that the research was conducted in the absence of any commercial or financial relationships that could be construed as a potential conflict of interest.
