# Supplementary material for: Computational implementation of a tunable multicellular memory circuit for engineered eukaryotic consortia
Source: Front Physiol. 2015 Oct 9;6:281. doi: 10.3389/fphys.2015.00281 (PMC4598587; doi:10.3389/fphys.2015.00281)
Supplement: Supplementary file 1 [file SupplementaryInformation.PDF]

Computational implementation of a tunable multicellular memory circuit for engineered eukaryotic consortia

Supplementary Information

Josep Sardanyés,[1,2] Adriano Bonforti,[1,2] Nuria Conde,[1,2] Ricard Solé*,[1,2,3] and Javier Macia †[1,2]

[1]*ICREA-Complex Systems Lab, Universitat Pompeu Fabra (UPF), Parc de Recerca Biomèdica de Barcelona (PRBB), Dr Aiguader 80, 08003 Barcelona, Spain*

[2]*Institut de Biologia Evolutiva, CSIC-UPF, Passeig Maritim de la Barceloneta, 37, 08003 Barcelona, Spain*

[3]*Santa Fe Institute, 1399 Hyde Park Road, Santa Fe NM 87501, USA*

(Dated: September 30, 2015)

* corresponding author

† corresponding author

I. QUANTITATIVE MATHEMATICAL MODEL

We built a mathematical model considering the cellular pathways and the interactions of four different engineered yeast cells. We specifically considered Cell 1 and Cell 2 (denoted as $C1$ and $C2$ in the model) responding to two different inputs, and Cells 3 and 4 (labeled $C3$ and $C4$ in the model) sensing two different types of α-factor molecules (wiring molecules). The wiring molecules are given, in a general way, by α-factor X (e.g., *Saccharomyces*) and Y (e.g., *Candida*). Based on the proposed cellular circuit displayed in Figure 1 of the main manuscript (see also Figure S1), we developed a mathematical model using ordinary differential equations as in (1). In the following lines we explain in detail the mathematical model. First, we introduce the equations for Cell 1 ($C1$) and Cell 2 ($C2$), given by:

Cell i (Ci), with ($i = 1, 2$):

$$\frac{d[MF\alpha\_\mathcal{S}_i\_mRNA\_Ci]}{dt} = K_i[input_i] - k_{31_{deg}}[MF\alpha\_\mathcal{S}_i\_mRNA\_Ci], \quad (1)$$

$$\frac{d[prepro\_\alpha\_\mathcal{S}_i\_Ci]}{dt} = k_{32}[MF\alpha\_\mathcal{S}_i\_mRNA\_Ci] - k_{33}[prepro\_\alpha\_\mathcal{S}_i\_Ci]. \quad (2)$$

Equation (1) describes the production of the α-factor mRNA and Eq. (2) models the synthesis of the prepro α-factor from the mRNA. The process is as follows. On one hand, Cell 1 responds to input R (e.g., galactose), which induces the synthesis of α-factor Y (e.g., *Candida*). Hence, $\mathcal{S}_1 = Y$ and $K_1 = K_R$. For input R we use available parameter values for galactose-mediated activation (see Table S2). On the other hand, Cell 2 responds to input S, producing α-factor X (e.g., *Saccharomyces*). Here, $\mathcal{S}_2 = X$ and $K_2 = K_S$ (see also Table S2). The prepro α-factor produced in each of these two cells is exported to the medium in the form of α-factor (see below).

The equations for Cells 3 and 4 ($C3$ and $C4$), are given by:

Cell j (Cj), with $j = 3, 4$:

$$\frac{d[Ste2\_\mathcal{S}_j\_Cj]}{dt} = \nu_{Ste2\_production} - k_{1\_j}[Ste2\_\mathcal{S}_j\_Cj] \times \times [\alpha\_\mathcal{S}_j\_in\_medium] + k_2[Ste2\_\mathcal{S}_j\_Ph\_Cj] - - k_4[Ste2\_\mathcal{S}_j\_Cj], \quad (3)$$

$$\frac{d[Ste2\_\mathcal{S}_j\_Ph\_Cj]}{dt} = k_{1\_j}[Ste2\_\mathcal{S}_j\_Cj][\alpha\_\mathcal{S}_j\_in\_medium] - - k_2[Ste2\_\mathcal{S}_j\_Ph\_Cj] - k_5[Ste2\_\mathcal{S}_j\_Ph\_Cj], \quad (4)$$

$$\frac{d[active\_Ste5complex\_Cj]}{dt} = k_6[inactive\_Ste5complex\_Cj][Ste2\_\mathcal{S}_j\_Ph\_Cj] - - k_7[active\_Ste5complex\_Cj], \quad (5)$$

$$
\begin{aligned}
\frac{d[inactive\_Ste5complex\_Cj]}{dt} &= -k_6[inactive\_Ste5complex\_Cj] \times \\
&\times [Ste2\_\mathcal{S}_j\_Ph\_Cj] + k_7[active\_Ste5complex\_Cj], \quad (6)\\
\frac{d[Fus3c\_Cj]}{dt} &= -k_{nuc\_imp}[Fus3c\_Cj] + k_{nuc\_exp}[Fus3n\_Cj]\frac{V_{nucleus}}{V_{cytoplasm}} \\
&- k_8[Fus3c\_Cj][active\_Ste5complex\_Cj] + \\
&+ k_9[Fus3ppc\_Cj], \quad (7)\\
\frac{d[Fus3ppc\_Cj]}{dt} &= k_{small} \cdot k_{nuc\_exp}[Fus3ppn\_Cj]\frac{V_{nucleus}}{V_{cytoplasm}} - \\
&- k_{nuc\_imp}[Fus3ppc\_Cj] + k_8[Fus3c\_Cj] \times \\
&\times [active\_Ste5complex\_Cj] - k_9[Fus3ppc\_Cj], \quad (8)\\
\frac{d[Fus3n\_Cj]}{dt} &= k_{nuc\_imp}[Fus3c\_Cj]\frac{V_{cytoplasm}}{V_{nucleus}} - k_{nuc\_exp}[Fus3n\_Cj] + \\
&+ k_{10}[Fus3ppn\_Cj], \quad (9)\\
\frac{d[Fus3ppn\_Cj]}{dt} &= -k_{small} \cdot k_{nuc\_exp}[Fus3ppn\_Cj] + k_{nuc\_imp}[Fus3ppc\_Cj] \times \\
&\times \frac{V_{cytoplasm}}{V_{nucleus}} - k_{10}[Fus3ppn\_Cj], \quad (10)\\
\frac{d[LacI\_mRNA\_Cj]}{dt} &= k_{34}[Fus3ppn\_Cj] - k_{LmRNA\_deg}[LacI\_mRNA\_Cj], \quad (11)\\
\frac{d[LacI\_Cj]}{dt} &= k_{Lac\_transl}[LacI\_mRNA\_Cj] - k_{Lac\_deg}[LacI\_Cj], \quad (12)\\
\frac{d[MF\alpha\_factor\_\mathcal{S}_p\_mRNA\_Cj]}{dt} &= \frac{\gamma}{1+\left(\frac{[LacI\_Cj]}{\beta_c}\right)^2} - k_{31_{deg}}[MF\alpha\_factor\_\mathcal{S}_p\_mRNA\_Cj], \quad (13)\\
\frac{d[prepro\_\alpha\_factor\_\mathcal{S}_p\_Cj]}{dt} &= k_{32}[MF\alpha\_factor\_\mathcal{S}_p\_mRNA\_Cj] - \\
&- k_{33}[prepro\_\alpha\_\mathcal{S}_p\_Cj], \quad (14)
\end{aligned}
$$

with $p, j = 3, 4$; and $p \neq j$. Here, Cell 3 is activated by the α-factor Y (i.e., $\mathcal{S}_3 = Y$), via the pheromone pathway (Eqs. (3)-(10) model the pheromone pathway, see (1)), which produces LacI that inhibits the synthesis of the other α-factor, given by the α-factor X (i.e., $\mathcal{S}_4 = X$). All circuit cells synthesise the prepro α-factor, which is processed and exported from the cells to the medium. The equations for the production of the alpha-factors X and Y in the medium are given by:

$$
\begin{aligned}
\frac{d[\alpha\_X\_in\_medium]}{dt} &= \frac{k_{33}([prepro\_\alpha\_X\_C2] + [prepro\_\alpha\_X\_C3])}{dilution} - \\
&- k_{\alpha\_Can\_deg}[\alpha\_X\_in\_medium], \quad (15)\\
\frac{d[\alpha\_Y\_in\_medium]}{dt} &= \frac{k_{33}([prepro\_\alpha\_Y\_C1] + [prepro\_\alpha\_Y\_C4])}{dilution} - \\
&- k_{\alpha\_Sac\_deg}[\alpha\_Y\_in\_medium]. \quad (16)
\end{aligned}
$$

TABLE S1: Initial conditions and ranges of input concentrations analysed. In all the analyses we allowed the system to evolve during 7 hours before the introduction of the first input in order to remove the transients, using the same initial conditions as in Ref. (1). For all of the simulations (including the exploration of the parameter space) we checked that the system achieved equilibrium before introducing the first input.

*Ranges of input concentrations analysed used to activate Cells 1 and 2.

[a] Different input concentrations are used to analyse the time to achieve the state after input introduction and the memory time.

Variables	Initial concentration ($nmol/ml$)	Molecule number per cell
$[Ste2\_S_p\_C3,4](0)$	378×10^{-3}	6600
$[Inactive\_Ste5complex\_C3,4](0)$	38.5×10^{-3}	672
$[Fus3n\_C3,4](0)$	568.4×10^{-3}	1390
$[Fus3c\_C3,4](0)$	406×10^{-3}	7090
Input	Concentration ($\mu g/ml$)	
Input R (e.g., Galactose)	$(0.005 - 500)$	$*^{a}$
Input S	$(0.005 - 500)$	$*^{a}$

Here, the dilution is the factor by which the α-pheromone is diluted when exported from the cells cytoplasm to the culture medium. This process, considering a constant cell population, can be modeled using $dilution = 13,800$ (1). The previous term assumes dilution in a cell culture density of 5×10^6 $cells/ml$. We notice that, as a difference from Ref. (1), we do not consider the growth of the population since dilution is kept constant, and we also introduce a parameter of elimination of the α-factors in the medium. This parameter describes the process of spontaneous decay or externally regulated removal of the pheromones from the medium e.g., by regulating an outflow in a microfluidic device (see Fig. 1d in the main manuscript).

In order to monitor the dynamics of our circuit in laboratory experiments, we must consider a reporter protein as output. Hence, we will also check if a green fluorescence protein (GFP) provides a good signal for the flip-flop behaviour. Since we are interested in monitoring the behaviour of these cells' output, the GFP gene can be added next to the α-factor gene (e.g., α-factor X or Y), being, the GFP gene, under the same promoter of the α-factor gene. To do so, we can add the to the equations of one of the Cells, $Cj = 3,4$, the dynamics of GFP production (1). Other constructions may be possible, but here we will focus on this particular case. Under this organisation, both α-factor and

GFP genes will be repressed by LacI (see Figure 1 in the main manuscript and Figure S1). The equations for this new block are given by (1):

$$\frac{d[GFP\_mRNA\_Cj]}{dt} = \frac{\gamma}{1+\left(\frac{[LacI\_Cj]}{\beta_c}\right)^2} - k_{34_{deg}}[GFP\_mRNA\_Cj], \tag{17}$$

$$\frac{d[nascent\_GFP\_Cj]}{dt} = k_{35}[GFP\_mRNA\_Cj] - k_{36}[nascent\_GFP\_Cj], \tag{18}$$

$$\frac{d[mature\_GFP\_Cj]}{dt} = k_{36}[nascent\_GFP\_Cj] - k_{GFP\_deg}[mature\_GFP\_Cj]. \tag{19}$$

The initial conditions used in the model are shown in Table S1. A description of the model parameters, their values or ranges, the reactions the model considers, as well as the sources from which the values were obtained are provided in Table S2.

1.1. Asymmetries in the production of α-factor

To introduce asymmetric production of one of the α-factors in the circuit, we modified Eqs. (1) for Cell 2 and Eqs. (13) for Cells 3, which produce α-factor X. The modified equations are now given by:

$$\frac{d[MF\alpha\_Sac\_mRNA\_C2]}{dt} = \mathcal{N} \cdot K_S[input_B] - k_{31_{deg}}[MF\alpha\_Sac\_mRNA\_C2], \tag{20}$$

and

$$\frac{d[MF\alpha\_factor\_Sac\_mRNA\_C3]}{dt} = \frac{\mathcal{N} \cdot \gamma}{1+\left(\frac{[LacI\_C3]}{\beta_c}\right)^2} - k_{31_{deg}}[MF\alpha\_factor\_Sac\_mRNA\_C3]. \tag{21}$$

Here we assume that the α-factor X is α-factor from *Saccharomyces*, and there is an $\mathcal{N}$-fold production of α-factor X relative to α-factor Y. This asymmetry could be relevant in the experimental implementation of the circuit since it is known that *Saccharomyces sp.* can contain multiple repeats of the mature pheromone sequence (2).

TABLE S2: Model parameters. The table displays (by columns) the parameter name, the description of the biological process tied to the parameter and the reaction labels (see Figure 2 in the main manuscript and Figure S1), the value or ranges and the units, and the source from which the parameter values have been obtained.

$^\diamondsuit$ For input R we will use available data for galactose.

§ This parameter may be asymmetric in a real experimental setup and will be explored separately (see Section S-III and Figure S6).

† Explored parameter ranges: $10^{-13} \leq k_S \leq 10^{-9.4}\ s^{-1}$; $10^{-8} \leq k_{34} \leq 10^{-3}\ s^{-1}$; $10^{-13} \leq \gamma \leq 10^{-8}\ mmol/(ml \cdot s)$; $10^{-13} \leq \beta_c \leq 10^{-8}\ mmol/ml$; and $10^{10} \leq k_{1\_Y} \leq 10^{12}\ ml/(mmol \cdot s)$.

♯ The mechanism encapsulating parameters $K_{R,S}$ will vary depending on the nature of the input. Generally, the input molecule will bind to its receptor triggering the activation of a given promoter. For instance, promoter $GAL1$ when we use galactose.

‡ Parameter ranges of GFP degradation rates used to show the equivalence between the time dynamics of GFP and the output of the Cells 3 and 4 (e.g., prepro α-factor X and Y).

*Data from *Escherichia coli.*

[a] Parameter used to analyse the time to achieve the desired ON or OFF state after input and the memory time.

[b] The transcription rates of the prepro α-factor mRNAs was fixed using available expression data for *E. coli.* Since this parameter is unknown for yeast (e.g., *Saccharomyces sp.* and *Candida sp.*), we will explore a wide range of values for these parameters, i.e., $10^{-13} \leq \gamma \leq 10^{-8}\ mmol/(ml \cdot s)$, also investigating asymmetries between both transcription rates.

[c] The elimination rate of both α-factors in medium was explored within the range $0.005 \leq k_{\alpha\_X,Y\_deg} \leq 500\ s^{-1}$, using the parameter values from this Table. The quality of the flip-flop was found to be maximum at $0.1 \lesssim k_{\alpha\_X,Y\_deg} \lesssim 0.275\ s^{-1}$, for a wide range of input values (Figures 5 and 9 in the main manuscript). Larger values of elimination, not considered in our model, gave place to poor or non existing flip-flop signals (results not shown).

Parameter name	Parameter description (*reaction label*)	Value and units	References
K_R	Promoter activation rate of $C1$ by input R (r_{Gal})	$1.2 \times 10^{-11}\ s^{-1}$	(3) $^\diamondsuit$ ♯
K_S	Promoter activation rate of $C2$ by input S (r_S)	$1.2 \times 10^{-11}\ s^{-1}$	§ † ♯
$k_{31_{deg}}$	Degradation rate of $MF\alpha1$-mRNA ($r_{31_{deg}}$)	$0.00231\ s^{-1}$	(4)
$k_{1\_X,Y}$	$Ste2 - \alpha$ binding rate (r_1)	$8 \times 10^{11}\ ml/(mmol \cdot s)$	(5) §
k_2	Release of α from Ste2 (r_2)	$3250\ s^{-1}$	(1)
$\nu_{ste2\_production}$	Ste2 synthesis rate (r_3)	$6.95 \times 10^{-12}\ mmol/(ml \cdot s)$	(5)

k_4	Ste2 degradation rate (r_4)	$1.84 \times 10^{-5}\ s^{-1}$	(1)
k_5	$Ste2-\alpha$ degradation rate (r_5)	$2.1 \times 10^{-5}\ s^{-1}$	(1)
k_6	Activation rate of Ste5 complex (r_6)	$18000\ ml/(mmol \cdot s)$	(1)
k_7	Inactivation rate of Ste5 complex (r_7)	$0.0042\ s^{-1}$	(1)
k_8	Phosphorylation rate of Fus3 (r_8)	$3.2 \times 10^{10}\ ml/(mmol \cdot s)$	(1)
k_9	Cytoplasmatic dephosphorylation rate of Fus3 (r_9)	$680\ s^{-1}$	(1)
k_{10}	Nuclear dephosphorilation rate of Fus3 (r_{10})	$0.28\ s^{-1}$	(1)
k_{nuc_imp}	Nuclear import rate of Fus3 and Fus3PP (r_{12}, r_{13})	$16.8\ s^{-1}$	(1)
k_{nuc_exp}	Nuclear export rate of Fus3 (r_{11}, r_{14})	$85.7\ s^{-1}$	(1)
k_{small}	Relation Fus3PP nuclear export to Fus3 export (r_{11})	0.5	(1)
k_{34}	Transcription rate of LacI mRNA from PFUS1 (r_{34})	$4 \times 10^{-6}\ s^{-1}$	(6) † [a]
k_{LmRNA_deg}	Degradation rate of LacI mRNA (r_{15})	$0.003167\ s^{-1}$	(7)* [a]
k_{Lac_transl}	Translation rate of LacI mRNA (r_{16})	$0.0475\ s^{-1}$	(8)*
k_{Lac_deg}	Degradation rate of LacI (r_{17})	$3.85 \times 10^{-4}\ s^{-1}$	(9)* [a]
β_c	LacI repression coefficient (r_{18})	$8 \times 10^{-9}\ mmol/ml$	(10)* † [a]
γ	Transcription rate of α-factor (r_{19})	$1.667 \times 10^{-8}\ mmol/(ml \cdot s)$	(10)* † [a b §]
$k_{\alpha\_X\_deg}$	Elimination rate of α-factor X in medium (r_{20})	$(0.005 - 200)\ s^{-1}$	(11) † [a c]
$k_{\alpha\_Y\_deg}$	Elimination rate of α-factor Y in medium (r_{21})	$(0.005 - 200)\ s^{-1}$	(11) † [a c]
k_{32}	α-factor preprotein synthesis rate (r_{32})	$3\ s^{-1}$	(12)
k_{33}	Processing and export of α-factor (r_{33})	$0.00315\ s^{-1}$	(6)
k_{34_deg}	Degradation rate of GFP-mRNA (r_{34_deg})	$0.00214\ s^{-1}$	(13)
k_{35}	GFP synthesis rate(r_{35})	$2\ s^{-1}$	(12)
k_{36}	GFP folding and maturation (r_{36})	$9.625 \times 10^{-5}\ s^{-1}$	(14)
k_{GFP_deg}	GFP degradation rate (r_{37})	$(0.00125 - 0.0025)\ s^{-1}$	(11) [‡]

II. TIMES RESPONSES TO ACTIVATION INPUTS AND MEMORY

In this Section we will investigate both the dependency of response and memory times of the circuit on the model parameters analysed in our work: $[input]$, k_{α_X,Y_deg}, γ, β_c, and k_{34}. Here, we will also follow the sequence of inputs: $(1,0)$, $(0,0)$, $(0,1)$, and $(0,0)$. The responses times indicate how long it takes to achieve the desired state (i.e., GFP ON or OFF) after activation inputs $(1,0)$ and $(0,1)$, while memory persistence indicates the time during which the outputs are maintained on an ON or OFF level (depending on the previous state) during memory inputs $(0,0)$. To compute

these characteristic times we define two thresholds (see dashed blue lines in Figure S3) that will be used to determine when a given state is achieved (during activation inputs $(1,0)$ or $(0,1)$) and how long it takes to leave the achieved state (memory time during $(0,0)$). In our computations we will use the time dynamics of the GFP in Cell 4 to characterise these times.

The effect of the input concentration on the response times is displayed in Figure S4a-b. Using the values from Table S2, the response times (black lines in Figure 7a) are shown to decrease for increasing input concentration, both in $(1,0)$ (solid line) and in $(0,1)$ (dashed line) inputs, although the circuit responds faster to input $(0,1)$. At increasing the input memory, the memory persistence increases in time, being longer during the second memory input $(0,0)$ (dashed line). One can note that when using the values of $\gamma = 10^{-13}\ mmol/(ml \cdot s)$ and $\beta_c = 10^{-11.75}\ mmol/ml$, both response and memory times largely improve. It is also worth to note that the effects of increasing input on response time and memory persistence are symmetrical.

The results concerning parameter k_{α_X,Y_deg} are provided in Figure S4c-d. The increase in the elimination rate of both α-factors above $k_{\alpha\_X,Y\_deg} \sim 0.134$ involves a longer time to achieve the expected state after input $(1,0)$, indicating that the response of the circuit becomes slower when the elimination rate increases. Both effects are especially important during activation input $(1,0)$ and during the first memory input $(0,0)$. The memory times are also impaired at increasing elimination rates, being shorter for larger values of elimination rates. These behaviours change when using the values of γ and β_c enhancing the quality of the flip-flop (see Figure S4d). Also in this case, both the response and the memory persistence times behave symmetrically as k_{α_X,Y_deg} increases.

Finally, we computed the response and the memory persistence times for γ, β_c, and k_{34}. As previously said, for lower values of γ the circuit response is enhanced (Figure S4e-f). Actually, under such values, the circuit is indeed in the expected state (ON state). Beyond $\gamma \sim 10^{-10.5}\ mmol/(ml{\cdot}s)$, response times and memory persistence times increase and decrease respectively during the first 15 hours (Figure S5e). These changes are not observed for the last 15 hours, when memory is longer maintained but the responses times are longer. The same simulations using $\beta_c = 10^{-11.75}\ mmol/ml$ reveal that the lowest responses times and the longer memories are also found for small values of γ, in agreement with all previous analyses showing that better qualities are achieved for low values of γ and β_c. The dependence of the responses time and the memory length for β_c can be found in Figure S5a-b, while for k_{34} the results are displayed in Figure S5c-d.

III. EFFECT OF CELLS ASYMMETRIES ON THE FLIP-FLOP BEHAVIOUR

In this section, in order to introduce further realism, we will consider asymmetries in some key parameters, as this may happen in real experiments. There are several reasons for asymmetric responses:

i) cell division taking place at different rates, depending on the genetic circuits introduced in each cell type, since different concentrations of cells for each cell type that introduce asymmetries in the production of α-factor; ii) different affinities of the $Ste2$ receptors of α-factor; and iii) different ratios of α-factor production for different cell types. Concerning point i), we introduce the asymmetry on the secretion of α-factor in Cells 1 and 2 due to the external inputs R and S by introducing the parameter ξ, which represents the relative efficiency of the secretion of α-factor in Cell 2 with respect to Cell 1. Values of $\xi < 1$ represents a lower efficiency on the secretion rate of α-factor in Cell 2 than in Cell 1 upon the same inputs concentrations. Oppositely, $\xi > 1$ indicates higher efficiency in Cell 2 than in Cell 1. Finally, $\xi = 1$ represents a symmetric system, as explored in the previous sections. Similarly, parameter η accounts for the relative efficiency of the $Ste2$ receptors to sense α-factor. Finally, the asymmetries associated with different secretion rates of α-factor are described by $\mathcal{N}$, which accounts for the relative secretion rates of α-factor Y with respect to α-factor X (see Section S1.1. for further details). In case of symmetrical production, $\mathcal{N} = 1$. One experimental case where $\mathcal{N} > 1$, would be given by the use of *Saccharomyces* and *Candida* α-factors, since *Saccharomyces cerevisiae* can contain multiple repeats of the mature pheromone sequence, so that, e.g., $\mathcal{N} = 4$ (2).

The effect of these asymmetries will be analysed using the values from Table S2 and values of γ and β_c giving a better flip-flop quality, as we previously characterised, i.e., $\gamma \sim 10^{-13}\ mmol/(ml \cdot s)$ and $\beta_c \sim 10^{-11.75}\ mmol/ml$.

The analyses for different values of ξ, using the values from Table S2 (solid line in Figure S6a), indicate that for values $\xi < 0.1$, the minimal conditions for flip-flop behaviour are not satisfied. At $\xi \sim 0.1$, the flip-flop quality increases significantly in a short range, the highest value being near the ratio $\xi \sim 0.5$, suggesting that a slight asymmetry on ξ might improve the system's behaviour. On the other hand, the results using the parameter values generating a good flip-flop quality show a constant value ($\Theta_S \sim 0.8$) for all values of ξ, indicating that the sensitivity of the response to external inputs has an important effect on the circuit behaviour (dashed line in Figure S6a). The dynamics of the circuit considering the asymmetry are displayed in panels (a.1) and (a.2) in Figure S6.

To investigate the impact of asymmetries in the $Ste2$ receptor, we computed the quality of the flip-flop for several values of η. Using values from Table S2, the minimal conditions for flip-flop behaviour are not satisfied under the value $\eta < 0.3$, as $\Phi(t)$ does not achieve negative values after input activation $(1, 0)$. For $\eta > 0.3$, we observed a rapid increase in quality, with a peak at $\eta \sim 1$, indicating that the symmetrical situation is optimal for these parameters (solid line in Figure S6b). Quality increases when lowering values of γ and β_c), showing a peak at $\eta \sim 1$ (dashed line in Figure S6b). Panels (b.1) and (b.2) in Fig. S9 display the dynamics for the analysed values of η for the two studied cases.

Finally, we explore scenario (iii), which considers asymmetries in the production of α-factors in

Cells 3 and 4. To do so, we modified the model equations by including a term that accounts for the increased production of α-factor X. The results using $1 \leq \mathcal{N} \leq 6$ are displayed in Figure S6c for both analyses using values from Table S2 and the good β_c and γ values. In both cases, better quality was found for the symmetric case $\mathcal{N} = 1$, which has been thoroughly studied in the previous sections. For the parameter values generating a good flip-flop behaviour, quality is highest for $\mathcal{N} = 1$ and remains high for any value of $\mathcal{N}$. For the simulations using the values from Table S2, the quality rapidly decreases by increasing $\mathcal{N}$, and the minimal conditions for flip-flop behaviour are lost for $\mathcal{N} > 3$. The time series for this case is shown in Panel (c.1). Interestingly, our simulations reveal that this negative effect can be avoided by decreasing the values of γ and β_c, which recovers the flip-flop shape and makes both the memory and responses to inputs $(1, 0)$ and $(0, 1)$ behave more correctly [see Panel (c.2) in Figure S6].

---

[1] Hoffman-Sommer,M., Supady,A. and Klipp,E. (2012) Cell-to-cell communication circuits: quantitative analysis of synthetic logic gates. *Front. in Physiol.*, **3**, 1-18.

[2] Brake,A.J., Julius,D.J. and Thorner,J. (1983) A functional prepro-alpha-factor gene in Saccharomyces yeasts can contain three, four, or five repeats of the mature pheromone sequence. *Mol. Cell. Biol.* **3**, 1440-1450.

[3] Li,J., Wang,S., van Dusen,W.J., Schultz,L.D., George,H.A., Herber,W.K., Chae,H.J., Bentley,W., E. and Rao,G. (2000) Green fluorescent protein in *Saccharomyces cerevisiae*; real-time studies of the GAL1 promoter. *Biotechnol. Bioeng.*, **70**, 187-196.

[4] Herrick,D., Parker,R. and Jacobson,A. (1990) Identification and comparison of stable and unstable mRNAs in *Saccharomyces cerevisiae*. *Mol. Cell. Biol.* **10**, 2269-2284.

[5] Yu,R., Pesce,C.G., Colman-Lerner,A., Lok,L., Pincus,D., Serra,E., Holl,M., Benjamin,K., Gordon,A. and Brent,R. Negative feedback that improves information transmission in yeast signalling. *Nature*, **456**, 755-761 (2008)

[6] Caplan,S., Green,R., Rocco,J. and Kurjan,J. (1991) Glycosylation and structure of the yeast MFα1 α-factor precursor is important for efficient transport through the secretory pathway. *J. Bacteriol.* **173**, 627-635.

[7] Bernstein,J.A., Lin,P-H., Cohen,S.N. and Lin-Chao,S. (2004) Global analysis of *Escherichia coli* RNA degradosome function using DNA microarrays. *Proc. Natl. Acad. Sci. U. S. A.* **101**(9), 2758-63.

[8] Bremer,H., Dennis,P.P. (1996) Modulation of chemical composition and other parameters of the cell by growth rate, in *Escherichia coli* and *Salmonella typhimurium*: Cellular and Molecular Biology, Neidhardt, Editor.

[9] Ceroni,F., Furini,S. and Cavalcanti,S. (2010) A computational model of gene expression in an inducible synthetic circuit. *Pacific Symposium on Biocomputing*, **15**, 409-420.

[10] Basu,S., Gerchman,Y., Collins,C.H., Arnold,F.H,. and Weiss,R. (2005) A synthetic multicellular system for programmed pattern formation. *Nature*, **434**(7037), 1130-1134.

[11] This parameter was unknown and was explored for the given ranges.

[12] von der Haar, T. (2008) A quantitative estimation of the global translational activity in logarithmically growing yeast cells. *BMC Syst. Biol.*, **2**, 87. dpi:10.1186/1752-0509-2-87.

[13] Hyde,M., Block-Alper,L., Felix,J., Webster,P. and Meyer,D.I. (2002) Induction of secretory pathway components in yeast is associated with increased stability of their mRNA. *J. Cell Biol.*, **56**, 993-1001.

[14] Heim,R., Cubitt, A.B. and Tsien,R. (1995) Improved green fluorescence. *Nature*, **373**, 663-664.

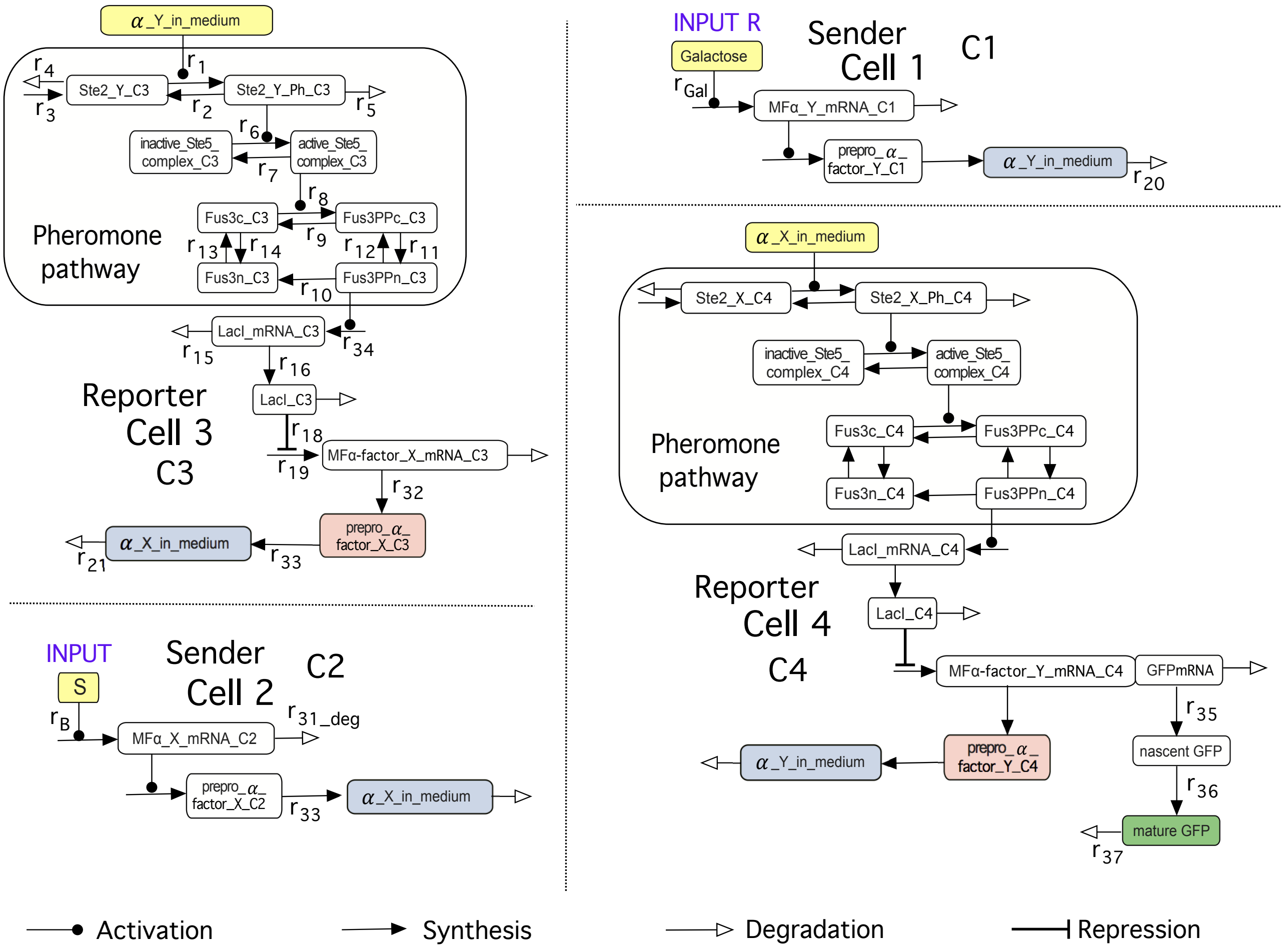

FIG. S1: Wiring diagrams displaying all the cellular pathways modeled with the mathematical model (see also Figure 1 in the main manuscript). We show the pathways for Cells 1 and 2. Cell 1 responds to the input R (e.g., Galactose) producing α-factor Y (e.g., *Candida*), while cell 2 responds to the input S synthesizing α-factor X (e.g., *Saccharomyces*). Cells 3 and 4 are activated by a different α-factor. They are engineered to behave as NOR gates, and contain LacI-mediated repression of each of the outputs. We also indicate the reactions considered by the model (see Table S2). The natural outputs of the circuit are given by the prepro α-factors X and Y (red boxes). The output can be also monitored by including a green fluorescent protein (GFP) in the cell (here included in Cell 4). The products taken from the medium are indicated in yellow boxes, while the products released by the cells to the medium are indicated with blue boxes.

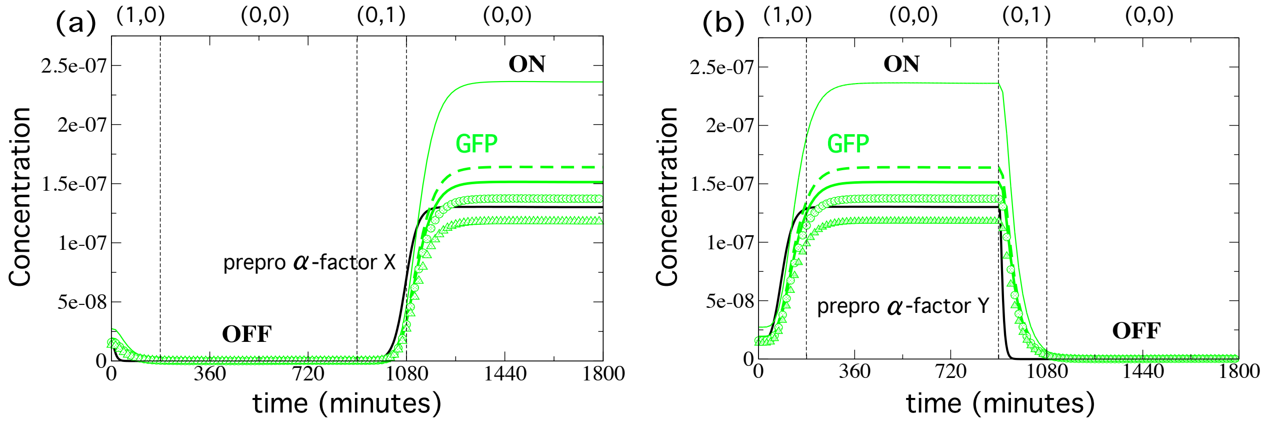

FIG. S2: Comparison of the outputs dynamics given by the prepro α-factors (black lines) and the green fluorescent protein (GFP, green trajectories) for Cell 3 (a) and Cell 4 (b). We used the values from Supplementary Table S2 except for $\gamma = 10^{-12.5}\ mmol/(ml \cdot s)$, $\beta_c = 10^{-11.8}\ mmol/ml$, with $[input] = 192\ \mu g/ml$, and $k_{\alpha\_X,Y\_deg} = 0.134\ s^{-1}$. In both panels we display different simulations for GFP with: $k_{GFP\_deg} = 0.00125\ s^{-1}$ (thin line); $k_{GFP\_deg} = 0.0018\ s^{-1}$ (thick dashed line); $k_{GFP\_deg} = 0.00195\ s^{-1}$ (thick solid line); $k_{GFP\_deg} = 0.00215\ s^{-1}$ ($\bigcirc$); and $k_{GFP\_deg} = 0.0025\ s^{-1}$ ($\triangle$). Notice that by tuning the degradation rate of the GFP the amplitude of the signal can be modulated without losing the flip-flop behaviour. This may be useful in experimental situations giving place to limited amplitudes of the flip-flop, which could be increased by decreasing GFP degradation rates, k_{GFP_deg}.

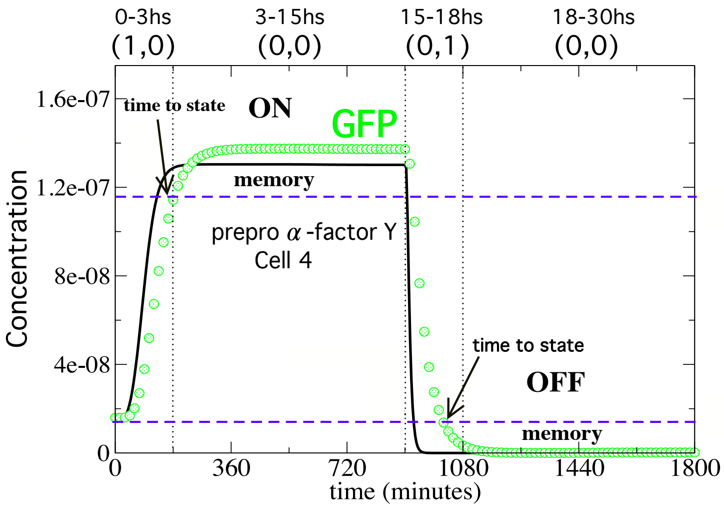

FIG. S3: Computation of the response dynamics (time it takes the circuit to achieve the desired ON or OFF state, indicated with black arrows) after activation inputs $(1, 0)$ and $(0, 1)$, as well as the memory time after memory inputs $(0, 0)$. These times are computed using two thresholds (dashed blue lines) to decide when the system achieves the ON or the OFF state, and how long the system remains in such states i.e., memory. Here we show an example for the reporter Cell 4, plotting the time dynamics of the prepro α-factor Y (black trajectory) and of the GFP (green trajectory). To compute these characteristic times we will monitor the dynamics of GFP, as we show in the figure.

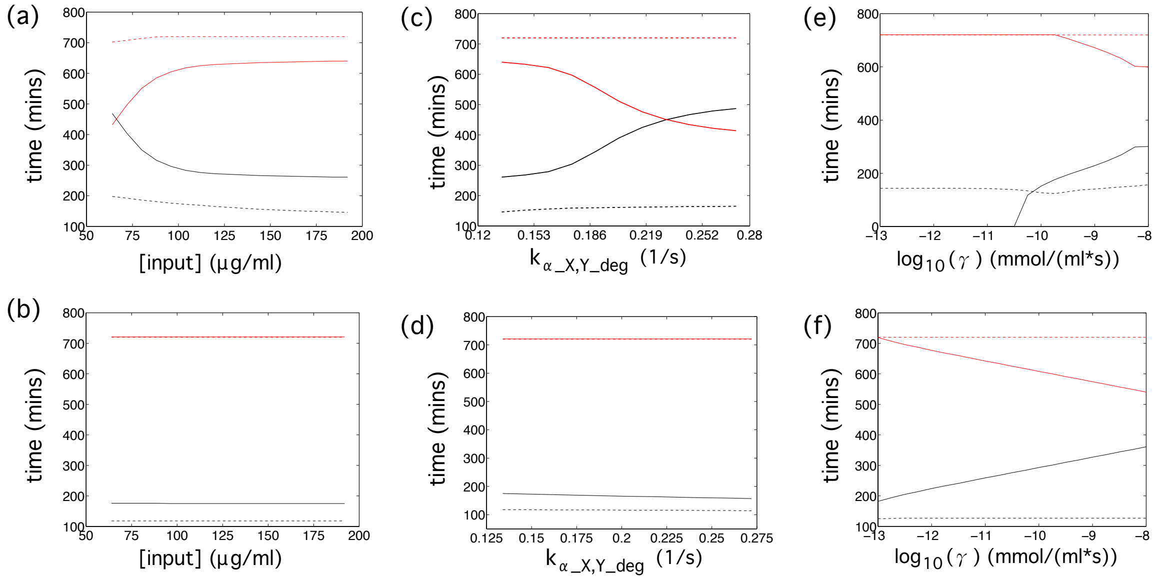

FIG. S4: Response time of the circuit and memory at increasing values of input concentration ($[input]$), elimination of alpha factor in medium (k_{α_X,Y_deg}), and transcription rate of α-factor (γ), computed from the time dynamics of the GFP produced by Cell 4. In (a), (c), and (e) we use the values form Supplementary Table S2. In (b) and (d) we set $\gamma = 10^{-13}$ $mmol/(ml \cdot s)$ and $\beta_c = 10^{-11.75}$, while in (f) we fixed $\beta_c = 10^{-11.75}$ $mmol/ml$. In all the plots we display the time that the circuit spends to achieve the desired state (response time indicated with black lines) after input $(1, 0)$ (solid line) and $(0, 1)$ (dashed line). In red we display the memory times after the first (solid line) and the second (dashed line) memory input i.e., $(0, 0)$, also following the series $(1, 0)$: 3 hours, $(0, 0)$: 12 hours, $(0, 1)$: 3 hours, and $(0, 0)$: 12 hours. In (a-b) and (e-f) we set $k_{\alpha\_X,Y\_deg} = 0.134$ s^{-1}, while in (c-f) we used $[input] = 192$ $\mu g/ml$.

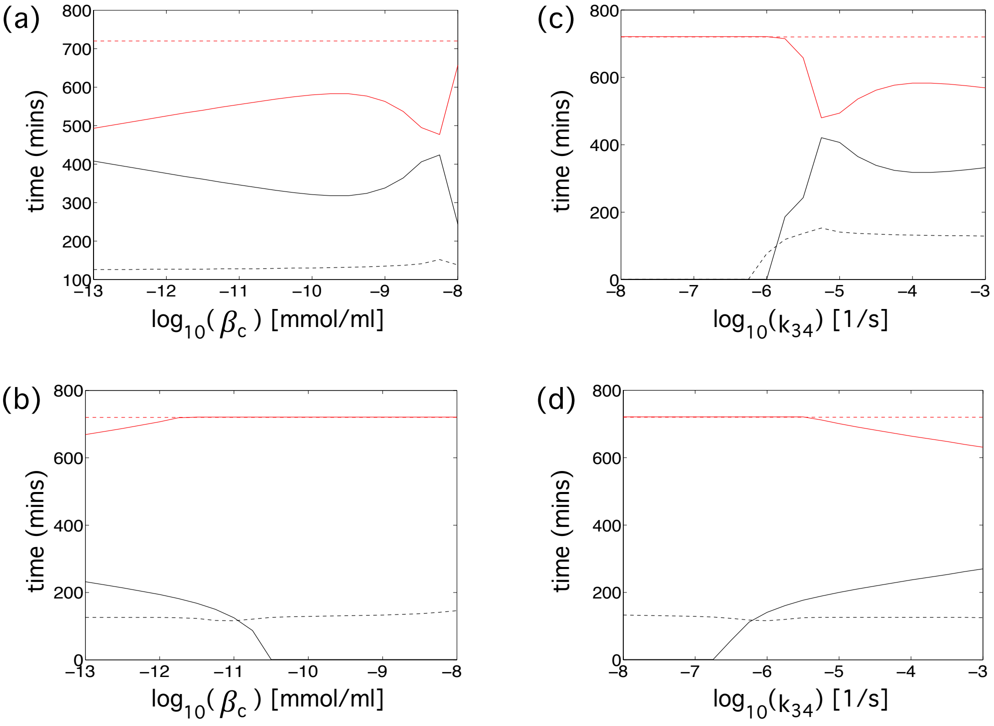

FIG. S5: Response time of the circuit and memory at changing values of the repression constant of LacI (β_c) and of transcription rate of LacI mRNA, k_{34} (also following the series $(1,0)$: 3 hours, $(0,0)$: 12 hours, $(0,1)$: 3 hours, and $(0,0)$: 12 hours). In (a) and (c) we use the values form Table S2, while in (b) and (d) we used values giving place to better flip-flop qualities, with $\gamma = 10^{-13}\ mmol/(ml \cdot s)$ (b); and $\gamma) = 10^{-13}\ mmol/(ml \cdot s)$ and $\beta_c = 10^{-11.75}\ mmol/ml$ (d). In all the plots we display the time that the circuit spends to achieve the desired state (response time indicated with black lines) after input $(1,0)$ (solid line) and $(0,1)$ (dashed line). In red we display the memory times after the first (solid line) and the second (dashed line) memory input i.e., $(0,0)$. In all the simulations we used $[input] = 192\ \mu g/ml$ and $k_{\alpha\_X,Y\_deg} = 0.134\ s^{-1}$.

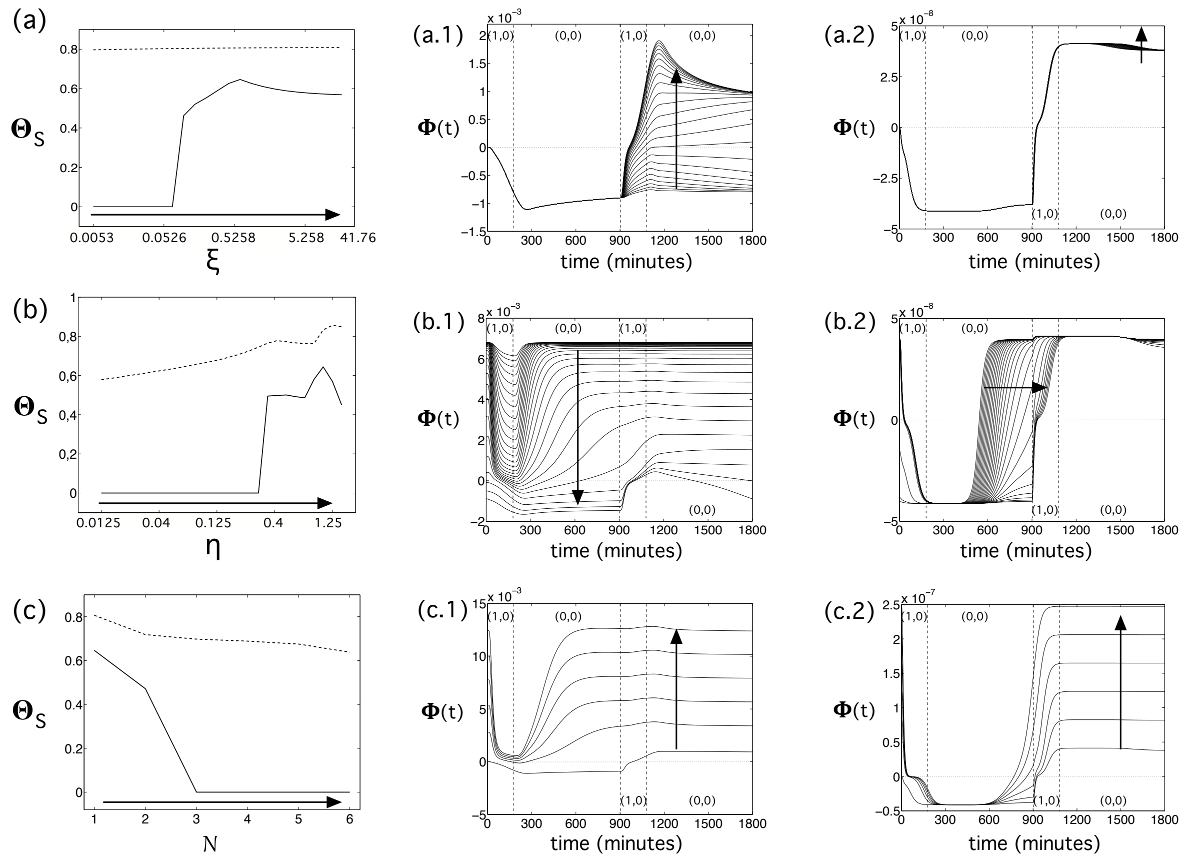

FIG. S6: Flip-flop quality, Θ_S, and dynamics considering asymmetries in the activation rates of the α-factor gene (represented with the ratio $\xi = K_S/K_R$) (a); in the affinities of the Ste2 receptor (represented with the ratio $\eta = k_{1,Y}/k_{1,X}$) (b); and considering $\mathcal{N}$-fold further production of prepro α-factor X (e.g., *Saccharomyces*) (c). For all of the parameter ranges analysed in (a-c), we used values from Table S2 (solid line) and values giving place to better qualities, with $\gamma = 10^{-13}\ mmol/(ml \cdot s)$ and $\beta_c = 10^{-11.75}\ mmol/ml$ (dashed lines). We also display the dynamics for all the parameter values analysed (indicated with the arrows), also using the values from Table S2 (1), and the γ and β_c values that produce better quality values (2). In all of the simulations, we also used $\lambda = 0.4$, $[input] = 192\ \mu g/ml$, and $k_{\alpha\_X,Y\_deg} = 0.134\ s^{-1}$.
